# The role of TOP2A in immunotherapy and vasculogenic mimicry in non-small cell lung cancer and its potential mechanism

**DOI:** 10.1038/s41598-023-38117-6

**Published:** 2023-07-05

**Authors:** Jiatao Wu, Lei Zhang, Wenjuan Li, Luyao Wang, Qianhao Jia, Fan Shi, Kairui Li, Lingli Liao, Yuqi Shi, Shiwu Wu

**Affiliations:** 1 Anhui Province Key Laboratory of Clinical and Preclinical Research in Respiratory Disease, Molecular Diagnosis Center, First Affiliated Hospital, Bengbu Medical College, 287 Changhuai Road, Bengbu, 233004 Anhui China; 2 Department of Pathology, The First Affiliated Hospital of Bengbu Medical College, Bengbu, People’s Republic of China; 3 Department of Oncology Surgery, The Second Affiliated Hospital of Bengbu Medical College, Bengbu, 233080 Anhui China; 4 Department of Radiation Oncology, The First Affiliated Hospital of Bengbu Medical College, Bengbu, 233000 Anhui China; 5 Department of Pathology, Bengbu Medical College, Bengbu, 233030 Anhui China; 6 Department of pathology, Anhui No.2 Provincial People’s Hospital, Bengbu, China

**Keywords:** Cancer, Computational biology and bioinformatics, Biomarkers, Oncology, Pathogenesis

## Abstract

Type IIA topoisomerase (TOP2A) is significantly associated with malignant tumor development, invasion, treatment and its prognosis, and has been shown to be a therapeutic target against cancer. In contrast, the role of TOP2A in the immunotherapy of non-small cell lung cancer as well as in Vasculogenic mimicry (VM) formation and its potential mechanisms are unclear. The aim of this study was to investigate the role of TOP2A in proliferation, skeleton regulation, motility and VM production in non-small cell lung cancer and its mechanisms by using bioinformatics tools and molecular biology experiments. Subgroup analysis showed that the low-risk group had a better prognosis, while the high-risk group was positively correlated with high tumor mutational load, M1-type macrophage infiltration, immune checkpoint molecule expression, and immunotherapy efficacy. As confirmed by further clinical specimens, the presence of TOP2A and VM was significantly and positively correlated with poor prognosis. Our study established a model based on significant co-expression of TOP2A genes, which significantly correlated with mutational load and immunotherapy outcomes in patients with non-small cell lung cancer. Further mechanistic exploration suggests that TOP2A plays an important role in immunotherapy and VM formation in NSCLC through upregulation of Wnt3a and PD-L1 expression.

## Introduction

Lung cancer is the leading cause of cancer-related deaths worldwide^1^, with an overall five-year survival rate of only 21%^2^. Non-small cell lung cancer (NSCLC) accounts for approximately 85% of all lung cancer patients, and lung adenocarcinoma (LUAD) and lung squamous cell carcinoma (LUSC) are the most common subtypes of non-small cell lung cancer. The mortality and aggressiveness of NSCLC are still uncontrollable, the metastasis and recurrence rates are high, and the tumor angiogenic activity is vigorous^3^.

Tumor angiogenesis is an independent prognostic factor for cancer patients because solid tumors are highly dependent on blood vessels for a continuous supply of oxygen and nutrients^3,4^. However, the clinical outcome of anti-angiogenic therapy at this stage is not as good as expected^5,6^, which may be due to our limited understanding of the vascular types and associated molecular mechanisms in NSCLC. Indeed, in addition to endothelium-dependent angiogenesis, Maniotis et al. identified for the first time a novel tumor blood supply pattern in human melanoma, namely VM^7^. VM is characterized by vascular-like structures formed directly by the arrangement of cancer cells and their extracellular matrix. The VM canal wall showed positive results for the special staining of periodic acid-Schiff (PAS) and negative results for endothelial cell markers such as CD31 and CD34. Vasculogenic mimicry is an important form of neovascularization found in highly aggressive tumors (including lung cancer), and it is closely related to the prognosis and metastasis of tumor patients^8–10^. In recent years, an increasing number of studies have focused on VM, but the specific mechanisms of VM formation remain unclear. At different stages of malignant tumorigenesis, invasive metastasis and angiogenesis are important factors contributing to tumor progression and malignant transformation^11,12^. Therefore, identifying and controlling the key proteins in the malignant process of tumors and understanding their regulatory mechanisms are of great practical importance to improve the survival of cancer patients.

Immune checkpoints are part of the body's immune system and exist to prevent the body from having an overactive immune response that injures normal cells. In contrast, tumor cells can induce immune checkpoint expression to evade immune destruction. When the T cell surface receptor PD1 binds to the tumor cell ligand PDL1, the T cells receive a "brake" signal and are not activated, resulting in the inability of the body's immune cells to recognize and clear the tumor cells^13–15^. The TIDE score is a response to sensitivity to immune checkpoint inhibitors and is used to evaluate immune evasion of tumors^16,17^. A higher TIDE score implies a higher likelihood of immune escape and a lower success rate of immunotherapy.

DNA topoisomerase II (TOP2A) is integrally associated with the development, invasion, treatment and prognosis of malignant tumors^18–21^. It regulates the biological behavior of tumors by participating in various biological processes such as cell cycle and apoptosis, and is closely related to the targets of chemotherapeutic agents for tumors. Although TOP2A has been shown to play a key role in tumors, it is still of practical importance to investigate the specific mechanisms of TOP2A's role in immune infiltration, immunotherapy and VM formation in non-small cell lung cancer.

It has been shown that WNT signaling contributes to endothelial cell differentiation and vascular remodeling, and that loss or gain of function of the WNT pathway may affect endothelial cell function, leading to abnormalities in vascular development and generation^22,23^. Wnt3a is composed of 19 cysteine-rich glycoproteins, which, as one of the classical WNT ligands, are closely associated with the etiology and pathology of several diseases, including cancer^24–26^. Previous studies have shown that through activation of the Wnt/β-catenin signaling pathway, Wnt3a can promote the epithelial-mesenchymal transition (EMT) process and enhance the metastatic properties of cancer cells^25,27,28^, both of which are closely associated with the formation of VM. Furthermore, direct correlation studies have shown that Wnt3a promotes the formation of VM in colorectal cancer^29^. Therefore, we hypothesize that Wnt3a plays an important role in the regulatory network of VM formation in NSCLC.

In this study, we constructed molecular subtypes of two TOP2A significantly co-expressed gene clusters using a bioinformatics approach and based on the GEO and TCGA databases of TOP2A significantly co-expressed genes. We further compared the differentially expressed genes (DEGs) between the two molecular isoforms and constructed a prognostic model for LUAD based on the DEGs. The final prognostic model was constructed by four genes together, TPX2, TOP2A, SFTBP and MYBL2. Further analysis showed that TPX2, TOP2A and MYBL2 were significantly upregulated in LUAD and their high expression was significantly and positively correlated with poor patient prognosis. The Tumor Immune Dysfunction and Exclusion (TIDE) also showed significant differences in the outcome of immunotherapy in patients with different types of LUAD. Subsequently, we analyzed the expression patterns of TOP2A, Wnt3a and VM in 141 cases of NSCLC using molecular biology experiments and further explored the intrinsic association among the three, TOP2A, Wnt3a and VM. Then, the effects of aberrant TOP2A gene expression in A549 and H1299 on the tubular structure formation ability, VM-related proteins, and immune checkpoint (PD-L1) expression in A5499 and H1299 cells cultured in vitro were investigated. In addition, we also used specific small interfering RNA to knock down the expression level of Wnt3a in overexpressed TOP2A cells and examined their tubular structure forming ability and VM-related protein expression to further verify the effect of TOP2A-Wnt/β-Catenin axis on VM formation. The final experimental results showed that TOP2A plays an important role in the malignant transformation of lung cancer through the upregulation of Wnt3a and immune checkpoint (PD-L1), suggesting the potential therapeutic value of targeting Wnt3a in inhibiting the progression and angiogenesis of non-small cell lung cancer.

## Results

### Screening for significant co-expression of TOP2A genes

Lung cancer-associated gene microarrays were obtained from GEO and TCGA datasets, and TOP2A significantly co-expressed genes (|R| > 0.6, *p* < 0.01, Supplementary Fig. 1A–C and Additional file 1: Table S2) were obtained from three microarrays, TCGA-LUAD, GSE19804, GSE116959, and 1111, 1194 and 247 genes were obtained, respectively. A total of 160 genes significantly co-expressed with TOP2A were obtained after plotting the Wayne diagram to take the intersection (Supplementary Fig. 1D and Additional file 1: Table S2).

### Consensus clustering to identify different subgroups and inter-cluster prognostic analysis

**Figure 1:**
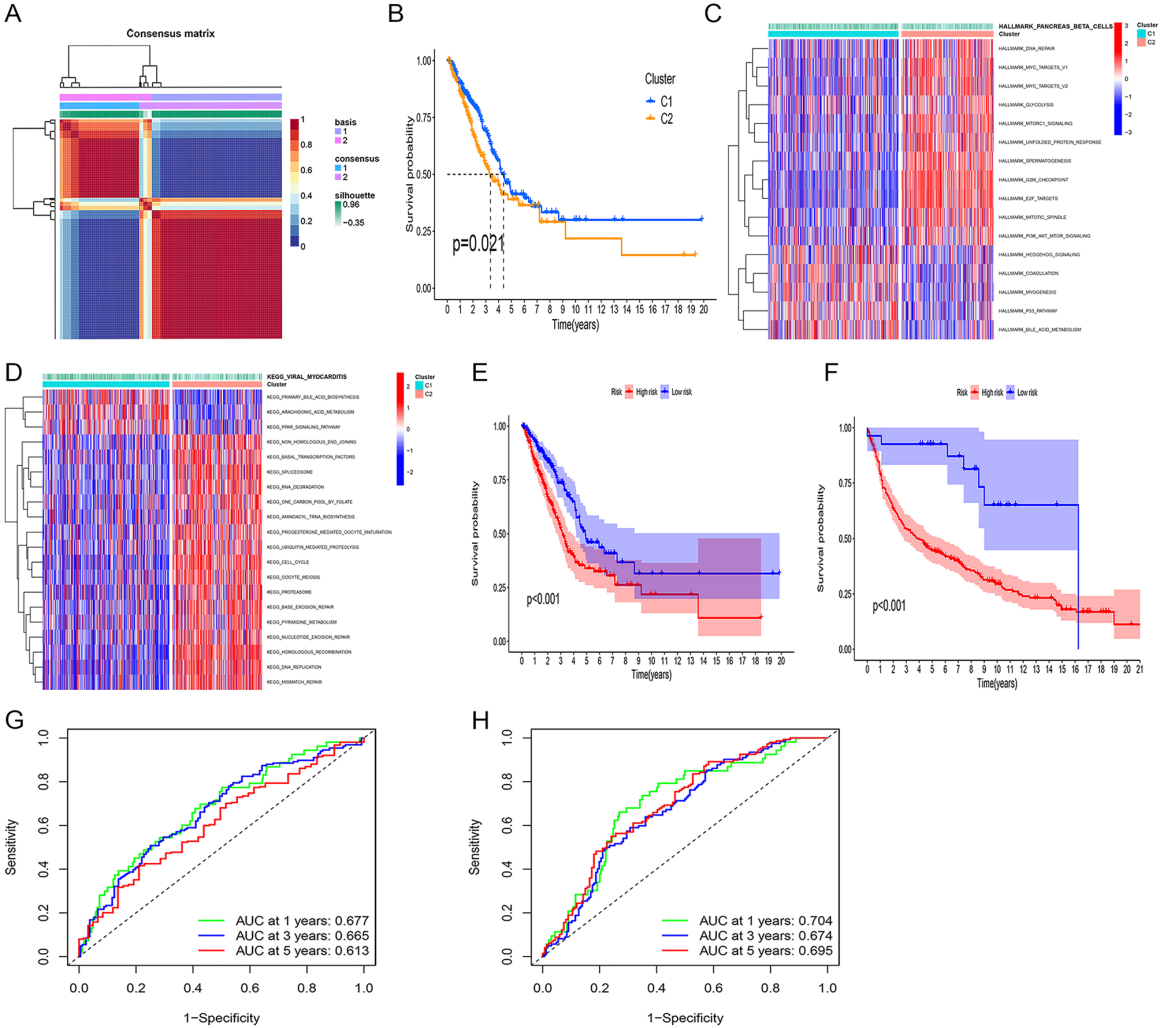
TOP2A co-expression patterns and associated biological pathways. (**A**) Non-negative matrix decomposition (NMF) clustering was performed and the best consensus clustering values for the two subgroups were determined. (**B**) Analysis of overall survival (OS) of 490 LUAD patients in the TCGA cohort based on two TOP2A co-expression clusters. (**C**,**D**) Heat map showing representative Hallmark and KEGG pathways in the two TOP2A co-expression clusters. (**E**,**F**) Kaplan–Meier curves for high and low TOP2A co-expression-associated gene subgroups of patients; (**E**) test cohort (TCGA-LUAD); (**F**) GEO cohort (GSE11969); (**G**,**H**) ROC curves showing TOP2A co-expression associated gene risk scores for predictive efficiency of 1-, 3-, and 5-year survival; (**G**) test cohort (TCGA-LUAD); (**H**) GEO cohort (GSE11969).

We analyzed the 161 significantly co-expressed genes of TOP2A including TOP2A obtained in TCGA-LUAD for differences (|logFC| > 0.6, p < 0.01), and a total of 83 genes with significant differences were obtained. Then, we merged the microarray data of LUAD gene expression with clinical data, and finally identified 490 LUAD samples with survival time for consistent clustering. Specifically, the expression of 83 TOP2A significantly co-expressed genes was decomposed into 2 non-negative matrices. The matrices were repeatedly decomposed and their outputs were aggregated to obtain consistent clustering of LUAD samples. The optimal number of clusters was selected based on the coeval coefficient, dispersion coefficient and profile coefficient. Consensus clustering was performed using the R package "NMF" (version 0.22.0) with the Blue Meta algorithm and 200 nruns (Fig. 1A and Supplementary Fig. 2A). The results of Kaplan–Meier survival analysis showed a significant difference between C1 and C2 (p < 0.05), and the samples in cluster 1 outperformed those in cluster 2 in terms of OS performance than those in cluster 2 (Fig. 1B). To clarify the difference in pathway expression between C1 and C2, we performed GSVA analysis using both HALLMARK and KEGG datasets, respectively, and the results showed that C2 with poorer prognosis was enriched in cell cycle pathway, mismatch repair pathway and some pathways that promote cell proliferation, and C1 with better prognosis was enriched in p53 pathway, PPAR pathway and HEDGEHOG pathway (Fig. 1C,D). The above results suggest that molecular typing of LUAD reconstructions based on significantly co-expressed genes of TOP2A is useful for predicting LUAD prognosis.

### Construction and identification of LUAD prognostic models based on significant co-expression genotyping of TOP2A

We first clarified the differences in gene expression profiles between the two subtypes C1 and C2, and a total of 25 differential genes were obtained (Additional file 1: Table S2). Using one-way Cox regression analysis, a total of 17 LUAD prognosis-related genes were obtained (Additional file 1: Table S2). A model for LUAD prognosis prediction was further developed using multifactorial Cox regression, and risk scores were determined by using the four most relevant genes. The risk score was calculated as follows: risk score = (1.972*TPX2 expression) + (− 0.804*TOP2A expression) + (− 0.719*MYBL23 expression) + (− 0.281*SFTPB expression). The training cohort or validation cohort samples were risk scored and ranked to determine whether expression levels changed systematically with risk score (Supplementary Fig. 2B,C). The expression levels of three of the genes increased significantly with increasing risk scores, whereas the opposite was true for SFTPB. In addition, there was a significant difference in prognosis between the two groups in either the training cohort or the validation cohort, with a significantly better prognosis in low-risk patients than in risk patients with a higher proportion of long-term survival (p < 0.001) (Fig. 1E,F). Results of the ROC curves in either the training cohort or the validation cohort showed prognostic prediction AUCs of 0.677, 0.665, and 0.613 at 1, 3, and 5 years for the internal data set, respectively; and 0.613; the prognostic prediction AUCs for 1, 3 and 5 years for the external data set were 0.704, 0.674 and 0.695, respectively, and the above results collectively indicate that the model has a good predictive effect (Fig. 1G,H).

### TCRGs signature predicts differences in tumor mutational burden and immune microenvironment

**Figure 2:**
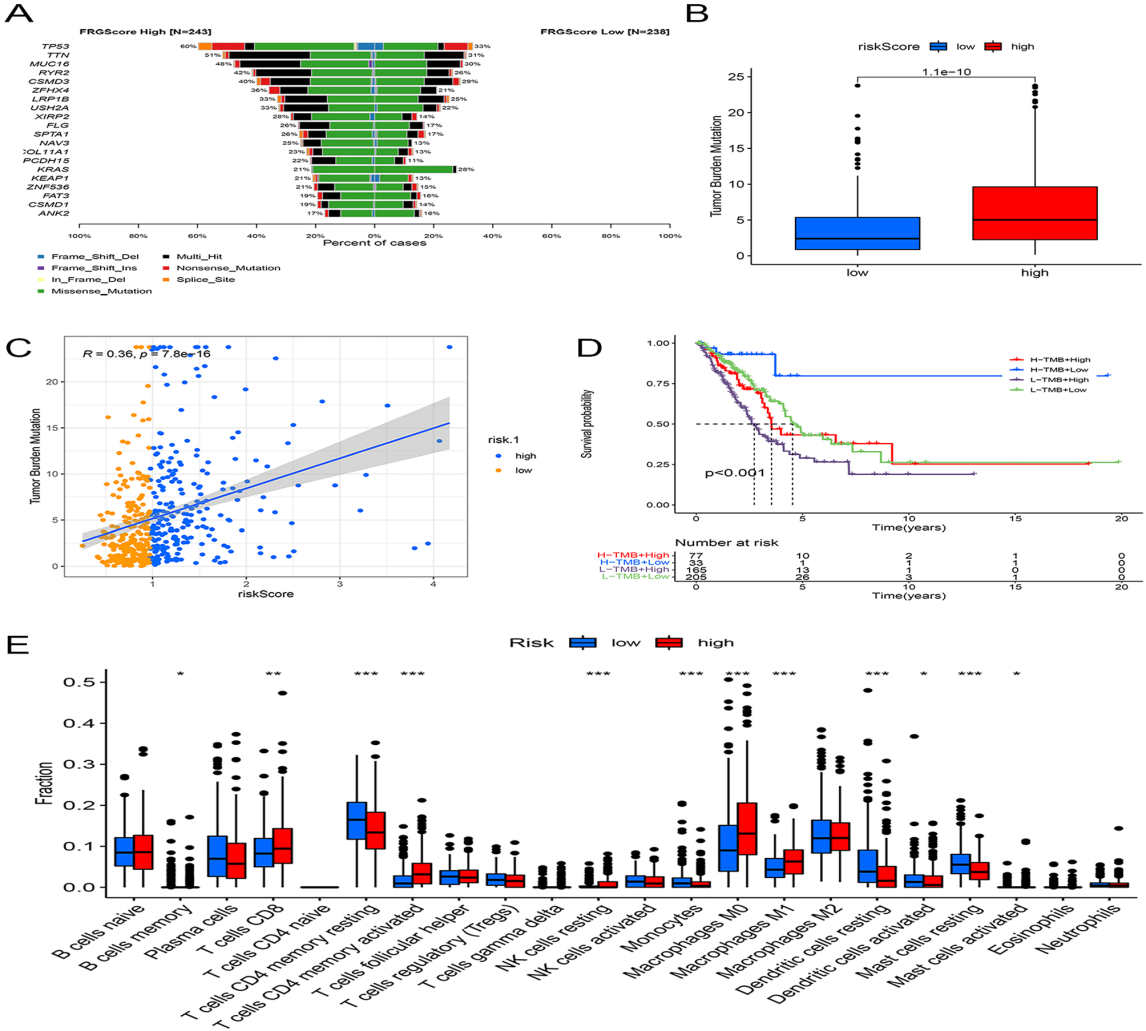
Relationship between TOP2A co-expression-associated gene risk cohort and LUAD tumor mutation burden and immune microenvironment. (**A**) Mutation profile of SMGs in TCGA-LUAD grouped by low and high TOP2A co-expression-associated gene scores. Each column contains a proxy for individual patients. (**B**) Relative distribution of tumor mutation burden in subgroups with high versus low TOP2A co-expression-associated gene scores. (**C**) Correlation between TMB and TOP2A co-expression-associated gene risk scores and their distribution in low- and high-risk groups. (**D**) Kaplan–Meier curves for high and low risk groups of TMB and TOP2A co-expression-associated genes. (**E**) Proportion of tumor-infiltrating immune cells in the high- and low-risk groups of TOP2A co-expression-associated genes using the CIBERSORT algorithm. In each group, scattered dots represent the expression values of tumor microenvironment cells. The thick line represents the median value. The bottom and top of the box are the 25th and 75th percentiles (interquartile range). Statistical differences were compared by Wilcoxon rank sum test as follows. *p < 0.05; **p < 0.01; ***p < 0.001.

Tumor mutational burden (TMB) is the total number of detected, somatic gene coding errors, base substitutions, gene insertion or deletion errors per million bases, and TMB has been found to be strongly associated with the prognosis and immune microenvironment of tumor patients. To clarify the relationship between TMB and immune microenvironment in LUAD, we first compared gene mutations in high- and low-risk cohorts, and the cross-sectional histogram showed a significantly higher mutation rate in the high-risk cohort than in the low-risk cohort (Fig. 2A), and a significantly higher number of TMBs in the high-risk cohort than in the low-risk cohort (p < 0.001, Fig. 2B). Further spearman correlation analysis showed a significant positive correlation between risk score and TMB (R = 0.36, p < 0.001, Fig. 2C). K-M survival curves demonstrated the survival status of LUAD patients in the TCEGs risk cohort combined with TMB, and the results showed that the survival prognosis of the low-risk cohort combined with high TMB was significantly higher than that of the high-risk cohort combined with low TMB survival prognosis (p < 0.001, Fig. 2D). Next, we further analyzed the correlation between the abundance of immune cell infiltration and the risk cohort, and the results showed that CD4+ memory dormant cells, monocytes, dormant and activated dendritic cells, and dormant mast cells were more enriched in the low-risk cohort, while M0 macrophages and M1 macrophages were more infiltrated in the high-risk cohort (Fig. 2E).

### Relationship between risk scores and immunotherapy

**Figure 3:**
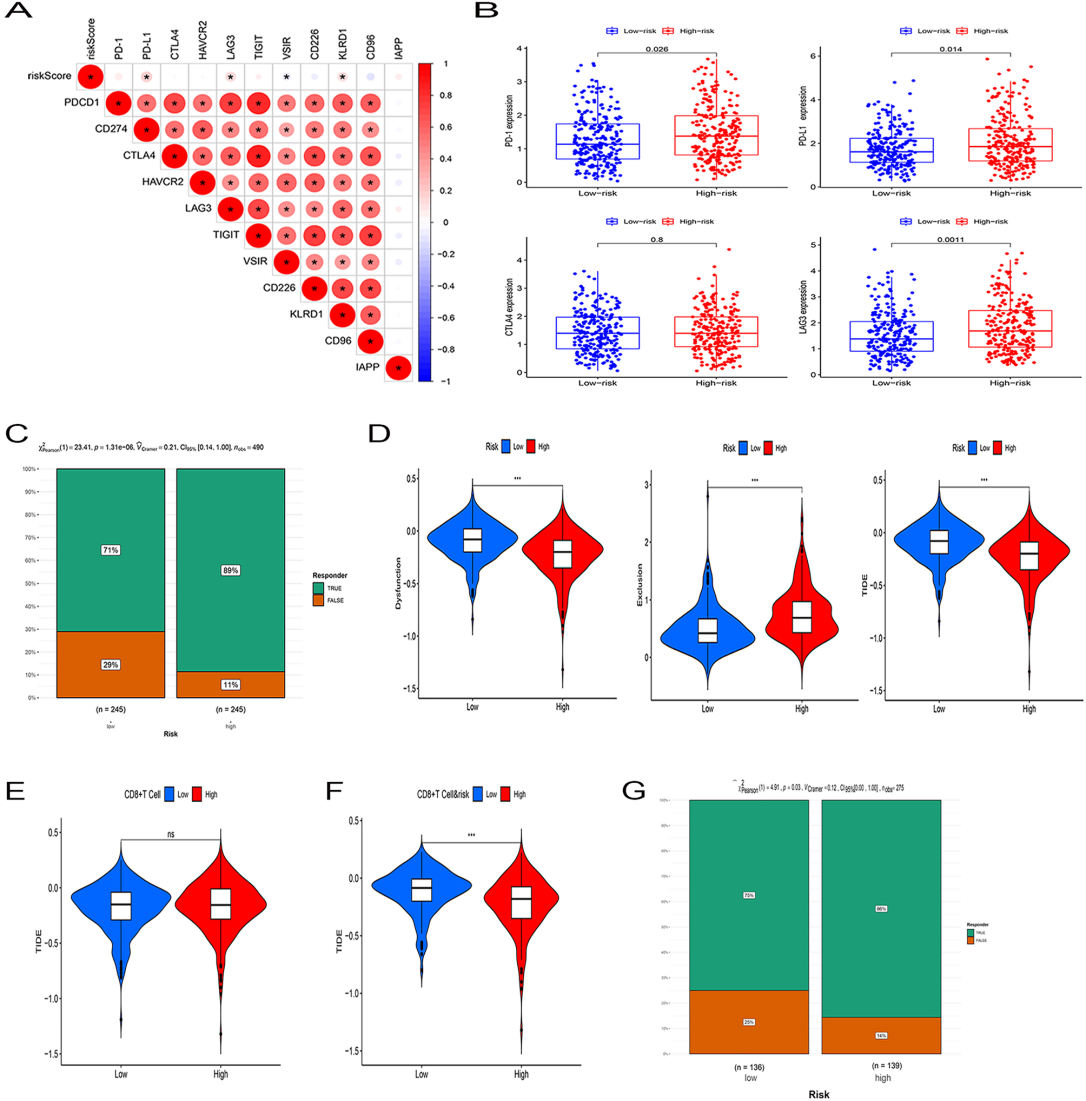
TOP2A co-expression-associated gene risk scores predict the efficacy of immunotherapy. (**A**) Association of TOP2A co-expression-associated gene risk scores with 11 immune checkpoints, with red circles representing positive associations and blue circles representing negative associations. (**B**) Distribution of four classical clinical immune checkpoint molecules in high and low risk of TOP2A co-expression-associated genes. (**C**) Proportion of patients with clinical response to anti-PD1 immunotherapy in the low or high TOP2A co-expression-associated gene score groups. CR/PR vs. SD/PD: 71% vs. 29% in the low VMRG score group; 89% vs. 11% in the high VMRG score group. (**D**) Functional distribution of immune dysfunction and immune escape in the high and low risk groups of TOP2A co-expression-related genes and the relative distribution of TIDE. (**E**) Relative distribution of TIDE in the high and low CD8+ T cell infiltration groups. (**F**) CD8+ T cell infiltration combined with high and low risk groups to analyze the relative distribution of TIDE. (**G**) CD8+ T-cell infiltration was integrated with high- and low-risk groups to analyze the proportion of patients with clinical responses to PD1 immunotherapy. CR/PR vs. SD/PD: 75% vs. 25% in the low-risk group; 86% vs. 14% in the high-risk group.

The expression of immune checkpoints affects the efficacy of LUAD patients, and to clarify the correlation between the two, we selected 11 immune checkpoint molecules and performed correlation analysis with risk scores, and the results showed a positive correlation between PD1, PD-L1 and LAG3 and risk scores (Fig. 3A,B). Next, we analyzed the proportion of immunotherapy efficacy in the risk cohort in the TIDE algorithm using a Chi-square test, and the results showed that treatment was effective in 71% and treatment was ineffective in 29% of the low-risk group, and treatment was effective in 89% and treatment was ineffective in 11% of the high-risk group (X^2^ = 23.41, p < 0.001, Fig. 3C). Immunodeficiency, immune escape and TIDE score ability of LUAD were further calculated using TIDE, and the results showed that immunodeficiency and TIDE score were higher in the low-risk group than in the high-risk group, while immune escape was higher in the high-risk group than in the low-risk group (Fig. 3D).

CD8+ T-cell infiltration is another good candidate for predictive immunotherapy. Therefore, we further calculated the TIDE score based on the abundance of CD8+ T-cell infiltration, and the results showed that there was no significant difference in the TIDE score between the two groups (Fig. 3E). However, when we further analyzed the TIDE score and the predicted effect of immunotherapy based on CD8+ T cell infiltration combined with high and low risk groups, we found that the TIDE score was lower in the high-risk versus CD8+ T-cell hyperinfiltrated group and the predicted effect of immunotherapy was significantly higher than that of the corresponding low-risk group (Fig. 3F,G).

### The four genes TPX2, TOP2A, MYBL23 and SFTPB in the TCEGs model can be regulatory targets of LUAD and are significantly associated with patient prognosis

We further evaluated the expression of the four genes TPX2, TOP2A, MYBL23 and SFTPB in the constructed TCEGs model in LUAD and the survival differences. The expression of TPX2, TOP2A and MYBL23 was significantly higher in cancer tissues than in paracancerous tissues, but SFTPB was lower in cancer tissues (Supplementary Fig. 3A–D), and that TPX2, TOP2A and MYBL23 high expression groups had poorer OS prognosis, while SFTPB high expression group had better OS (p < 0.05, Supplementary Fig. 3E–H). Meanwhile, the prognosis of PFS was poorer in the TPX2 high expression group, while PFS was better in the SFTPB high expression group (p < 0.05, Supplementary Fig. 3I–L). The above results suggest that the three genes TPX2, TOP2A and MYBL23 in the TCEGs model can be used as potential therapeutic targets to regulate the development of LUAD.

### TOP2A regulates the proliferative capacity of non-small cell lung cancer

**Figure 4:**
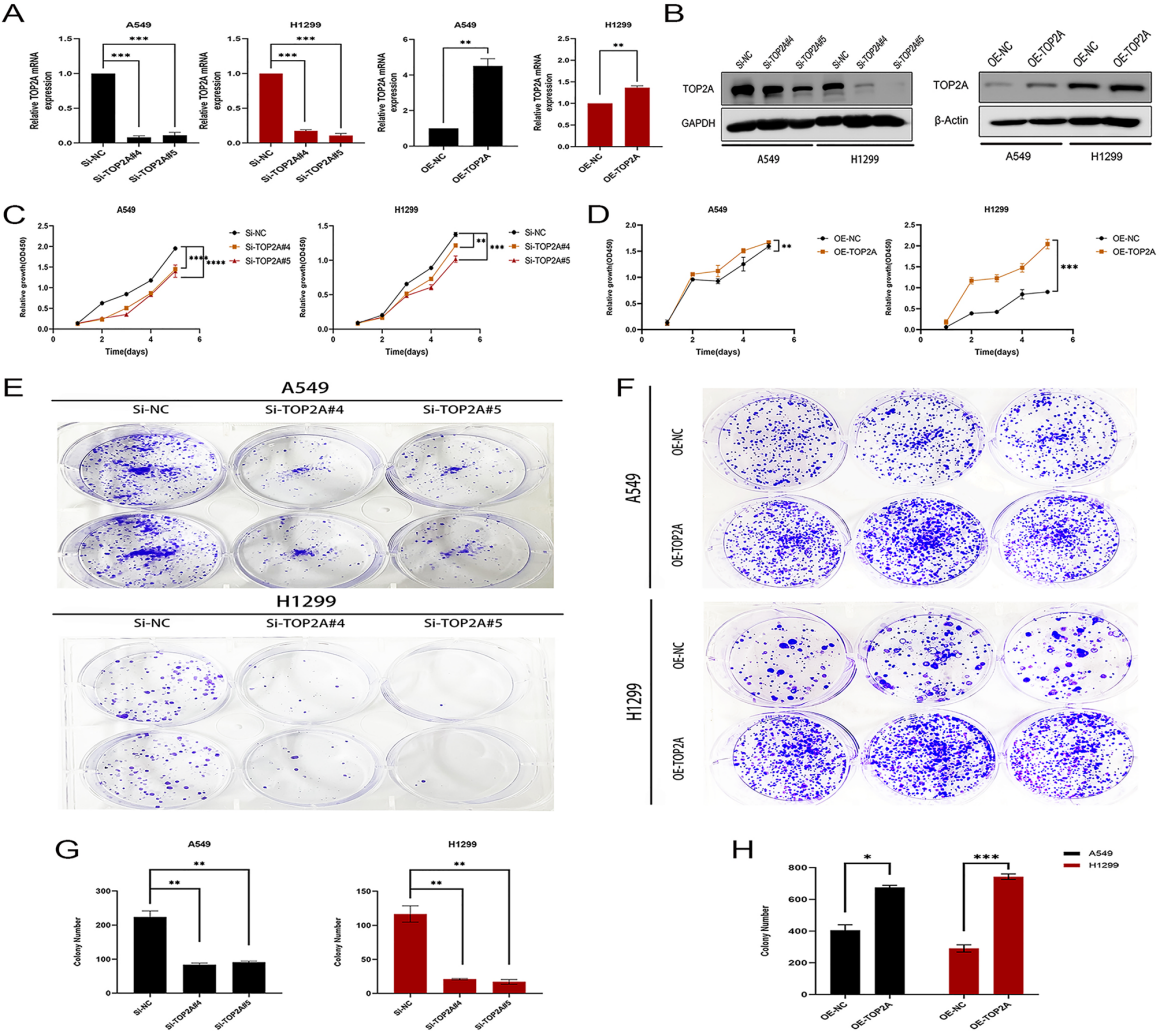
TOP2A regulates the proliferative capacity of non-small cell lung cancer. (**A**,**B**) Stable overexpression and knockdown systems of TOP2A identified by WB and qPCR. Some blot strips were cut prior to hybridization with the antibody, and the original blot strips are listed in the supplemental material (Supplementary Original western blot images). (**C**,**D**) Proliferation capacity of A549 and H1299 cells after overexpression or knockdown of TOP2A was detected by CCK-8 assay. (**E**–**H**) Colony formation analysis of non-small cell lung cancer cell growth. Data were determined by three replicate experiments (mean ± standard deviation).

To investigate the biological function of TOP2A in non-small cell lung cancer, we first constructed NSCLC cell lines with stable high expression of TOP2A in A549 and H1299 cells using a lentiviral vector carrying the full-length gene of TOP2A, and established TOP2A-silenced cell lines in A549 and H1299 cells using two specific siRNAs. After transfection for 48 h, qRT-PCR and Western blotting analysis showed that compared with the control, TOP2A expression was significantly increased in A549-TOP2A and H1299-TOP2A cells, while TOP2A expression was significantly decreased in A549-siTOP2A#4, A549- siTOP2A#5, H1299-siTOP2A#4 and H1299-si TOP2A#5 cells (Fig. 4A,B). The proliferation ability of tumor cells was detected by CCK8 assay, and the growth curves showed that knockdown of TOP2A significantly inhibited the proliferation ability of A549 and H1299, while the proliferation ability of A549 and H1299 was significantly enhanced when the intracellular TOP2A expression level was increased (Fig. 4C,D). The regulatory effect of TOP2A on the proliferation ability of NSCLC cells was further confirmed by colony formation assays, which showed that knockdown of TOP2A gene significantly inhibited the clonogenic ability of NSCLC cells (Fig. 4E,G), while the clonogenic ability of NSCLC cells overexpressing TOP2A was significantly enhanced (Fig. 4F,H).

### TOP2A regulates apoptosis and cell cycle in non-small cell lung cancer

**Figure 5:**
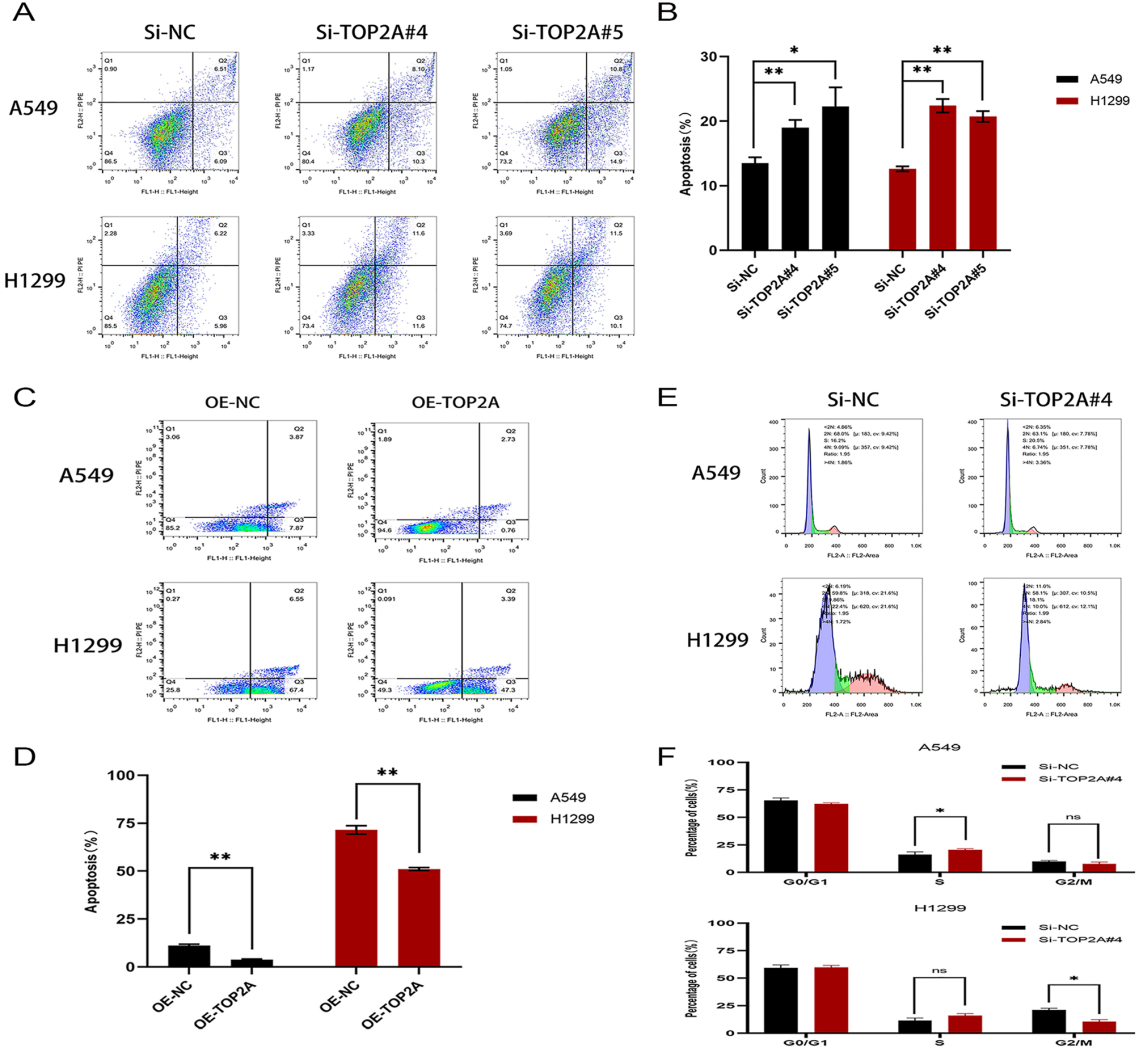
TOP2A regulates apoptosis and cycle in non-small cell lung cancer. (**A**,**B**) Flow cytometry to detect apoptosis after A549 and H1299 transfection with Si-TOP2A. (**C**,**D**) Flow cytometry to detect apoptosis after A549 and H1299 transfection with OE-TOP2A. (**E**,**F**) Flow cytometry to detect the cell cycle of A549 and H1299 after transfection with Si-TOP2A.

We further validated the role of TOP2A in regulating apoptosis and cycle in non-small cell lung cancer using apoptosis and cell cycle assays. The results of apoptosis assay revealed that when TOP2A was knocked down by specific siRNA, apoptotic cells were significantly increased in the TOP2A knockdown group compared with the negative control group (Fig. 5A,B), while the apoptosis rate of cells was significantly and significantly decreased when TOP2A was in the overexpression state (Fig. 5C,D). The results of cell cycle experiments showed that there was a significant S-phase block in the cell cycle when TOP2A was specifically knocked down by siRNA (Fig. 5E,F). All the above results indicated that TOP2A plays an important role in the development of non-small cell lung cancer and regulates the biological behavior of non-small cell lung cancer.

### TOP2A expression affects PD-L1 expression and VM formation in non-small cell lung cancer

**Figure 6:**
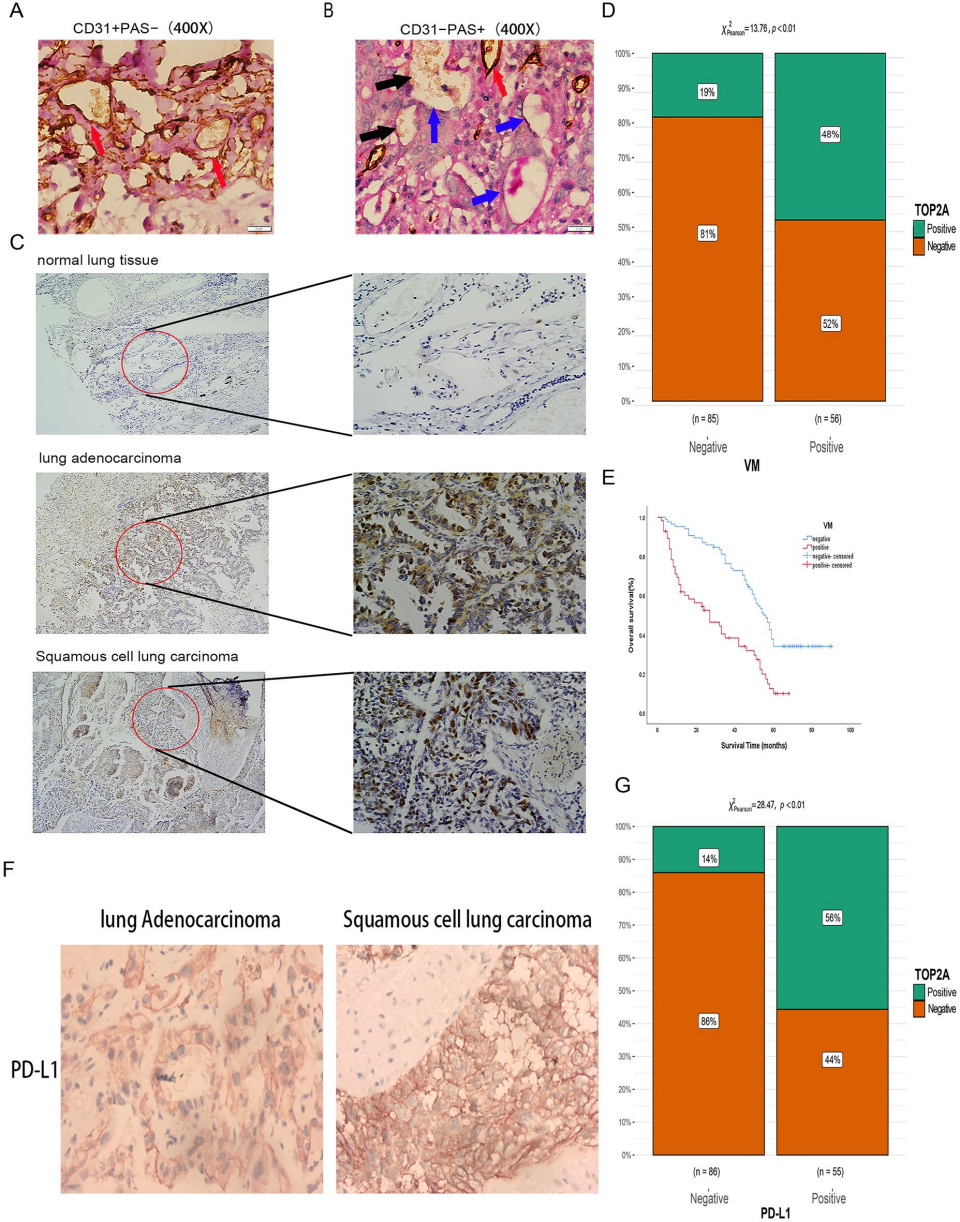
TOP2A expression was significantly correlated with PD-L1 expression and VM formation in NSCLC tissues. (**A**) The presence of VM was detected by CD31/PAS double staining. CD31+PAS− channels are endothelium-dependent vessels (red arrows). (**B**) CD31−PAS+ channels are considered VM (blue arrows), in which erythrocytes are visible (black arrows). (**C**) Typical NSCLC adjacent to normal and tumor tissue specimens with representative TOP2A staining (400 ×). (**D**) Immunohistochemical methods to detect VM expression in TOP2A-positive and TOP2A-negative groups. (**E**) Kaplan–Meier survival analysis showed poor overall survival in patients with high VM expression. (**F**) Representative PD-L1 immunohistochemical staining in lung adenocarcinoma and lung squamous carcinoma (200 ×). (**G**) Detection of PD-L1 expression in TOP2A-positive and TOP2A-negative groups by immunohistochemistry.

VM channels were reported to be CD31-negative and PAS-positive. Based on this pathological feature, we found the presence of VM ducts in 56 of 141 NSCLC specimens (Fig. 6A,B). To assess the effect of TOP2A on VM duct formation, we further examined TOP2A expression in 141 matched pairs of NSCLC specimens of tumor origin and adjacent non-tumor tissue origin by immunohistochemistry (Fig. 6C). The results showed that the expression level of TOP2A was significantly higher in non-small cell lung cancer tissues than in matched paraneoplastic tissues, with 56 of 141 non-small cell lung cancer specimens, while 27 of these 56 VM-positive specimens expressed TOP2A, but none of the 69 TOP2A-negative specimens expressed VM ductal structures. Upon further statistical analysis, there was a significant difference in the appearance of VM ductal structures in the TOP2A-positive group (VM-positive rate: 48%) and the TOP2A-negative group (VM-positive rate: 19%) (p < 0.01, Fig. 6D). Finally, further clinicopathological analysis of patients with high VM expression and Kaplan–Meier survival analysis showed that high VM expression was significantly and positively correlated with poorer tumor stage as well as tumor metastasis in patients (p < 0.05, Additional file 1: Table S3), and patients with high VM expression had poorer overall survival (p < 0.05, Fig. 6E).

Meanwhile, we also analyzed the effect of TOP2A expression on PD-L1 expression, and the results showed that there was a significant difference in the PD-L1 positivity rate between the TOP2A positive group (PD-L1 positivity rate: 56%) and the TOP2A negative group (PD-L1 positivity rate: 14%) (p < 0.01, Fig. 6F,G). In conclusion, TOP2A expression was significantly and positively correlated with PD-L1 expression.

### Exogenous TOP2A regulates VM formation, cell plasticity and motility

**Figure 7:**
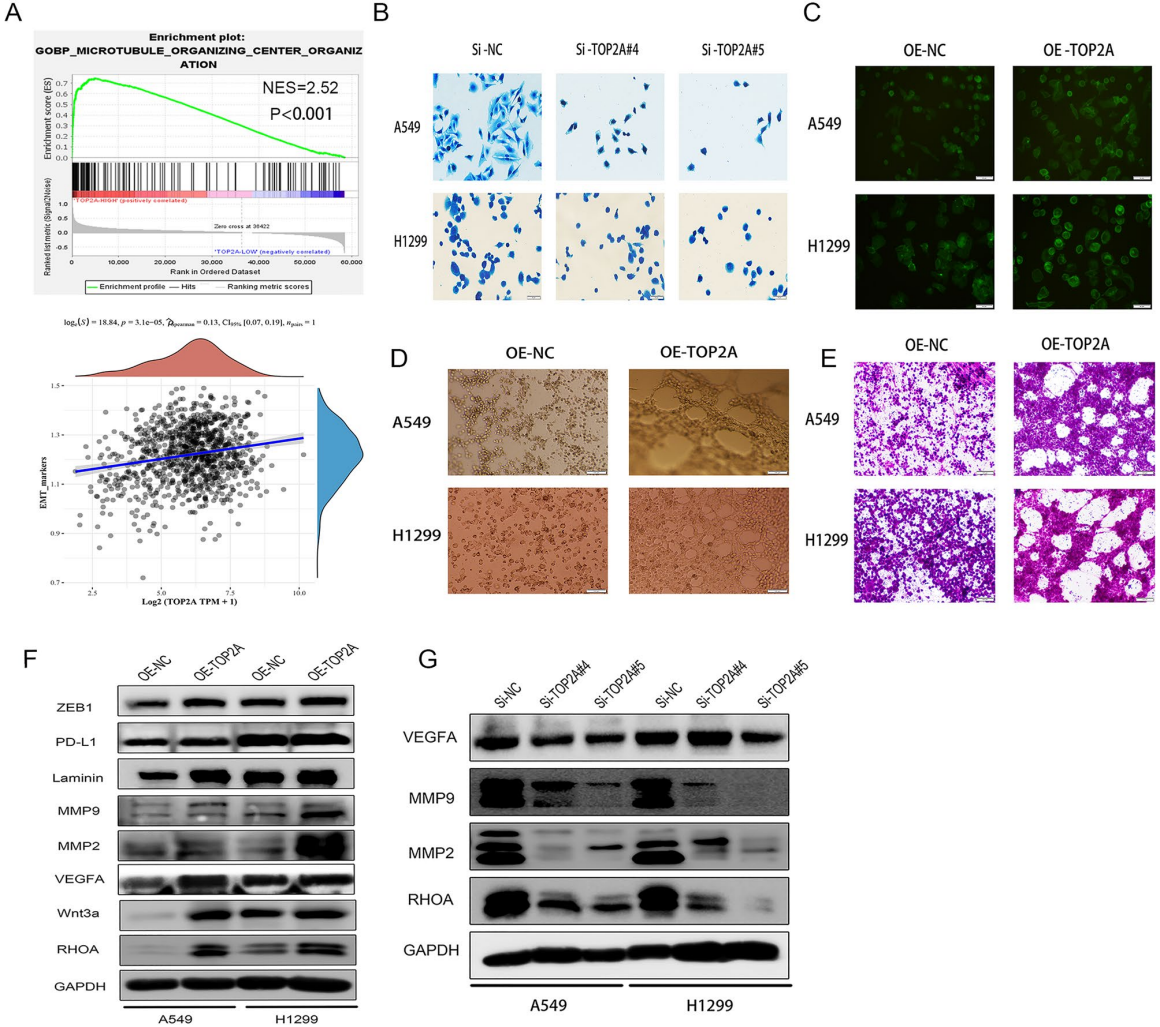
TOP2A is involved in regulating the cytoskeleton of NSCLC and VM formation in A549 and H1299 cells in vitro. (**A**) The relationship between EMT and TOP2A expression was analyzed by TCGA-LUAD (n = 465). (**B**) The effect of TOP2A knockdown on the cytoskeleton of A549 and H1299 cells was detected by Comas Brilliant Blue staining. (**C**) FITC-ghost pen cyclic peptide immunofluorescence assay the effect of overexpression of TOP2A on the cytoskeleton of A549 and H1299 was detected. (**D**,**E**) Tube formation assay showed that overexpression of TOP2A promoted the formation of VM in A549 and H1299 cells. PAS staining of vascular-like structures in Matrigel showed a significant decrease in the total area and total length of tube formation in the TOP2A overexpression group. (**F**,**G**) Western blotting was performed to detect the expression of VM formation-related proteins in A549 and H1299 cells. Some blot strips were cut prior to hybridization with the antibody, and the original blot strips are listed in the supplemental material (Supplementary Original western blot images).

To further investigate the role of TOP2A in non-small cell lung cancer angiogenesis, we explored the intrinsic connection between TOP2A expression pattern and tumor cytoskeleton and motility using GSEA based on GEO and TCGA databases. The results showed that biological processes such as cytoskeleton and cell motility were significantly enriched in the TOP2A high expression group (Fig. 7A, Supplementary Table 4). Tumor cells possessing plasticity and migration ability were more likely to form VM tubular structures^30–34^. Immunofluorescence assay with FITC-ghost pencil cyclic peptide and Comas Brilliant Blue staining showed that when TOP2A was knocked down, tumor cells exhibited contracted and disordered stress fibers, while when TOP2A was overexpressed, the cytoskeleton of tumor cells was remodeled and expression was enhanced (Fig. 7B,C).

Further tube-forming experiments with cells grown on Matrigel showed that human non-small cell lung cancer cell lines A549 and H1299 had poor tube-forming ability in vitro, whereas both cell lines showed a significant increase in the total tubular area and total tubular length in stromal gel when stimulated by exogenous TOP2A (Fig. 7D). Subsequently, PAS staining of the formed VM network and quantitative analysis of the total tube formation area and total tube length showed that the VM formation ability of A549 and H1299 cells overexpressing TOP2A was significantly enhanced (Fig. 7E).

Aberrant expression of VM production-related proteins (MMP2, MMP9, VEGFA, Laminin, Wnt3a, RHOA) as well as immune checkpoint (PD-L1) in tumors and the occurrence of EMT process are considered to be important drivers of VM formation and tumor development as well as metastasis. Therefore, we further assessed the expression of VM generation-related proteins, immune checkpoint (PD-L1) and ZEB1, a marker of EMT occurrence, using Western blot. We found that the expression of VM generation-related proteins MMP2, MMP9, VEGFA, Laminin, cytoskeleton regulatory proteins RHOA, Wnt3a, EMT occurrence marker ZEB1 and immune checkpoint (PD-L1) were significantly upregulated in cells overexpressing TOP2A compared with control cells (Fig. 7F). In contrast, the expression of VM generation-related proteins MMP2, MMP9, VEGFA and cytoskeleton regulatory protein RHOA were significantly down-regulated after TOP2A knockdown (Fig. 7G).

### Wnt3a knockdown counteracts the effects of TOP2A on plasticity and motility as well as VM formation in A549 and H1299 cells

**Figure 8:**
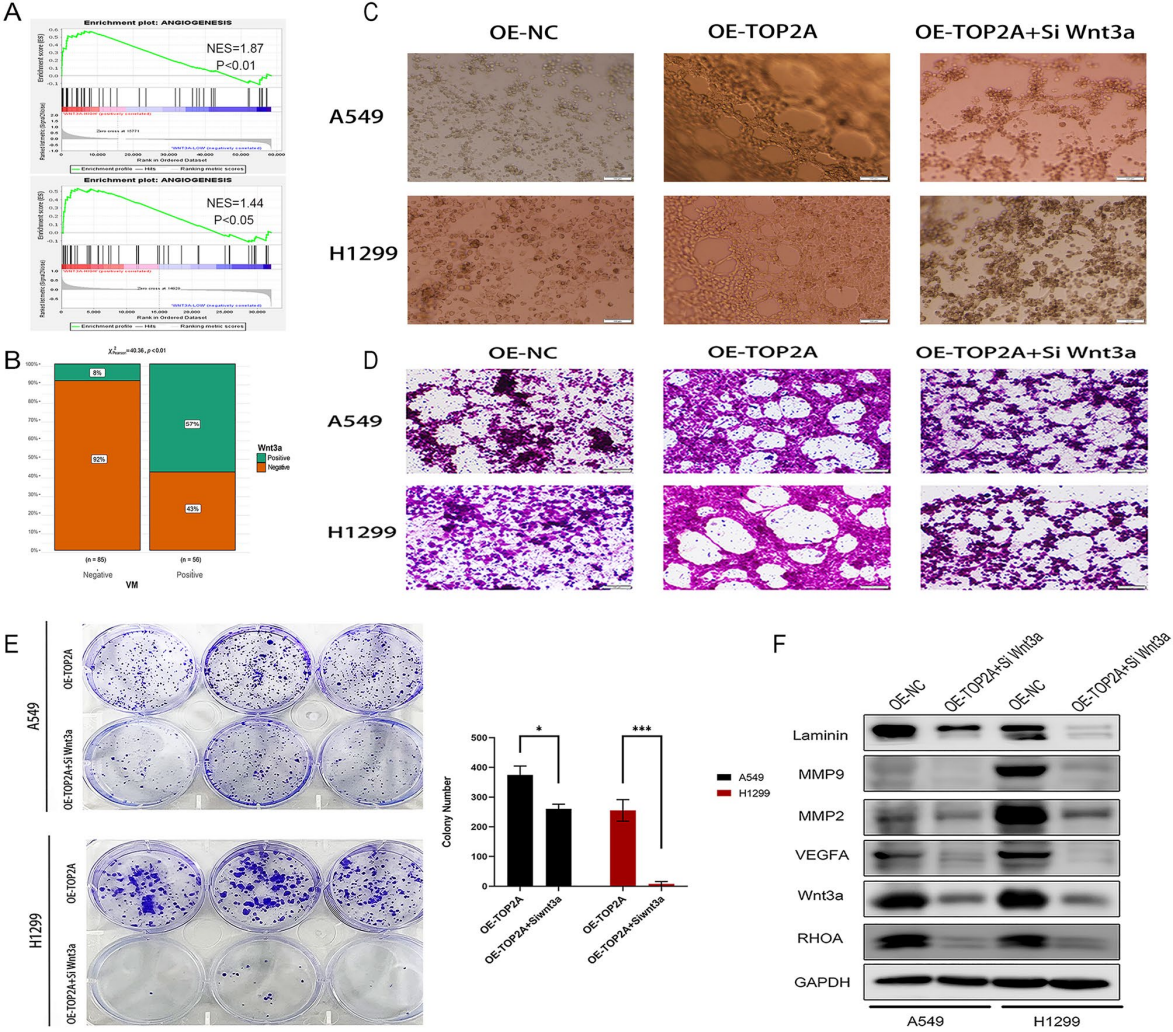
Knockdown of Wnt3a counteracted the TOP2A-induced invasive phenotype. (**A**) Enrichment analysis of TCGA (n = 585) and GSE116959 (n = 68) revealed that the angiogenic signaling pathway was significantly enriched in the Wnt3a high expression group. (**B**) Immunohistochemical methods to detect VM expression in the Wnt3a positive and Wnt3a negative groups. (**C**,**D**) The results of tube formation assay showed that Wnt3a knockdown significantly inhibited the VM formation ability of A549 and H1299 cells compared with the TOP2A overexpression group. (**E**) Wnt3a knockdown significantly inhibited the colony formation ability of non-small cell lung cancer cells compared with the control group. (**F**) Western blot detection of VM in overexpressed TOP2A cells before and after knockdown of Wnt3a expression of formation-related proteins. Some blot strips were cut prior to hybridization with the antibody, and the original blot strips are listed in the supplemental material (Supplementary Original western blot images).

It has been shown that Wnt3a promotes VM formation in colorectal cancer and breast cancer^29,35^. Therefore, we hypothesized that Wnt3a may play an important role in the regulatory network of VM formation in NSCLC. Firstly, we found that the angiogenic pathway was significantly activated in the Wnt3a overexpression group using a bioinformatics approach (Fig. 8A). Further correlation analysis of Wnt3a and VM expression in clinical specimens by immunohistochemistry revealed that 56 of 141 non-small cell lung cancer specimens expressed VM, while 32 of 56 VM-positive specimens had Wnt3a expression, while none of 78 Wnt3a-negative specimens expressed VM. The expression of VM was found to be significantly different between the Wnt3a positive and Wnt3a negative groups (p < 0.01, Fig. 8B). Further 3D culture results showed that both A549-TOP2A and H1299-TOP2A significantly promoted the formation of tube-like structures compared to the control group. However, when Wnt3a was silenced, even though TOP2A was in the overexpressed state, it still resulted in partial loss or disruption of tubular structures in the stromal gel, and both cell lines showed a significant decrease in the total tubular area and total tubular length in the stromal gel (Fig. 8C,D).

The results of further colony formation experiments also showed that the clone-forming ability of NSCLC cells was significantly enhanced after TOP2A overexpression; however, when the Wnt3a gene was knocked down, it counteracted the TOP2A-induced NSCLC clone-forming ability (Fig. 8E). Meanwhile, as expected, the TOP2A-induced expression of MMP2, MMP9, VEGFA, Laminin and RHOA all showed a corresponding down-regulation trend after the Wnt3a gene was knocked down (Fig. 8F).

## Discussion

TOP2A is a DNA topoisomerase that plays a crucial role in a variety of malignancies, including NSCLC^18,36^. However, its role in immunotherapy and angiogenic mimetic formation in NSCLC and its specific regulatory mechanisms remain unclear. In the current study, we provide evidence that TOP2A is involved in regulating VM production as well as immune checkpoint (PD-L1) expression in NSCLC and promotes VM formation in NSCLC both in vitro and in vivo. Furthermore, our data suggest that TOP2A may regulate cell plasticity, motility, and VM pipeline structure formation by affecting the expression of the classical ligand protein Wnt3a and the skeleton regulatory protein RHOA in NSCLC cell lines. Therefore, targeting Wnt3a and VM has potential value for the treatment of NSCLC.

In the present study, we found VM passage patterns in 56 of 141 NSCLC patients (39.7%). Previously reported results showed that the incidence of VM was 28.5%, 34%, 33.3%, 19.2%, 22.7% and 26.9% in gastric cancer, cutaneous melanoma, interstitial esophageal cancer, colorectal cancer, osteosarcoma and non-small cell lung cancer, respectively^37–42^. Interestingly, VM is present in less than 50% of almost all cancer types examined to date, and the researchers suspect that VM may be present only in subphenotypes of invasive or malignant cancers^43,44^. This could explain why the formation of VM usually predicts a poor prognosis for cancer patients. In the clinical specimens we collected and collated, the expression of TOP2A and Wnt3a was significantly increased in NSCLC tissue samples compared to the corresponding normal tissues. It has been shown that TOP2A and Wnt3a play an essential role in the development of NSCLC and tumor metastasis, and the expression of TOP2A and Wnt3a is closely related to tumor differentiation, TNM stage and the occurrence of lymph node (LN) metastasis^45–47^. On this basis, we further analyzed the correlation between the expression of TOP2A and Wnt3a and the presence of VM ducts in NSCLC, and found that the presence of VM ducts was observed in approximately 48% and 57% of NSCLC with positive expression of TOP2A and Wnt3a, respectively, while only 19% and 8% of tumors with negative expression of TOP2A and Wnt3a, respectively, could be observed. The presence of VM ducts was observed in only 19% and 8% of TOP2A and Wnt3a negative tumors, respectively. Pathological studies showed that NSCLC tissues overexpressing TOP2A and Wnt3a were prone to the formation of VM structures. Taken together, TOP2A and Wnt3a may play an important role in VM formation and cancer progression in NSCLC.

In recent years, immune checkpoint inhibitors (ICIs) have been shown to be one of the most promising and effective immunotherapies, which reconstitute anti-tumor responses and prevent tumor cells from evading immune surveillance by targeting specific molecules, such as programmed death receptor 1 (PD-1) or its ligand (PD-L1) and cytotoxic T-lymphocyte antigen 4 (CTLA-4)^48–50^. Our current findings suggest that TOP2A is involved in regulating the expression of immune checkpoint (PD-L1) in NSCLC, and the upregulation of TOP2A significantly promotes the expression of immune checkpoint (PD-L1). Further in vitro experiments also highlighted the critical role of TOP2A in VM channel formation, and TOP2A upregulation significantly promoted the skeletal remodeling of A549 and H1299 cells and the ability to form VM ducts in Matrigel gel. In our further study, expression of VM generation-related proteins MMP2, MMP9, VEGFA and cytoskeleton regulatory protein RHOA was significantly decreased in TOP2A gene silenced H1299 and A549 cells, while TOP2A upregulation significantly promoted VM generation-related proteins MMP2, MMP9, VEGFA, Laminin, Wnt3a, the expression of cytoskeleton regulatory protein RHOA and EMT occurrence marker ZEB1. On the other hand, the presence of VM ductal structures was detected in 57% of Wnt3a-positive NSCLC tissues, whereas the presence of VM channels was detected in only 8% of Wnt3a-negative samples, and further knockdown of Wnt3a expression in H1299 and A549 cell lines overexpressing TOP2A significantly inhibited their formation of VM ductal structures in vitro, so we believe that Wnt3a is essential in the formation of VM in NSCLC. Taken together, all of the above results further validate our hypothesis that TOP2A may regulate cell plasticity, motility and ultimately promote the formation of VM ductal structures by affecting the expression of the classical ligand protein Wnt3a and the cytoskeleton regulatory protein RHOA in NSCLC cells.

In conclusion, the current study links TOP2A overexpression, plasticity and motility of non-small cell lung cancer cells, immunotherapy, and the formation of VM. Our study suggests that Wnt3a expression potentially provides an accurate marker for the formation of VM ductal structures in patients. Indeed, Wnt3a is involved in several processes, including tumor cell proliferation, migration, and tumor angiogenesis^24,51–53^. Therefore, we believe that Wnt3a is an excellent antitumor target and future work should also consider Wnt3a as a potential marker for VM channels in non-small cell lung cancer.

## Conclusions

TOP2A plays an important role in regulating immunotherapy and VM formation in non-small cell lung cancer by regulating the expression of the classical WNT ligand Wnt3a and the immune checkpoint PD-L1. This study highlights the potential therapeutic value of targeting Wnt3a to inhibit NSCLC progression and VM generation.

## Methods

### Collect and preprocess publicly available expression datasets

Gene expression data and clinical characteristics of NSCLC samples were collected from publicly available datasets from the NCBI GEO database and TCGA database. A total of 922 patients were included in the analysis, including the GSE11969 (N = 149)^54^, GSE19804 (N = 120)^55^, GSE116959 (N = 68)^56^, and TCGA-LUAD (N = 585) datasets. Since these GEO datasets share the same microarray sequencing platform (AffymetrixHG-U133 + 2.0), we downloaded the raw "CEL" files and processed them with the "affy" and "Simple Processing" packages. Bulk effects between different GEO datasets were removed using the ComBat method in the "SVA'R" package. Genomic mutation data for TCGA-LUAD were obtained from the UCSC Xena database and Davoli et al. Non-synonymous mutations (including shift mutations, missense mutations, nonsense mutations and splice site mutations) counts were identified as tumor mutation burden (TMB).

### Consensus molecular clustering of TOP2A significantly co-expressed genes by NMF

Based on the expression levels of the significantly co-expressed genes of TOP2A, LUAD patients were classified into different molecular subtypes using "NMF". The optimal number of clusters was selected based on the co-expression coefficient, dispersion coefficient and profile coefficient. Consensus clustering was run 200 times using the R package "NMF" (version 0.22.0) with the Blue Element algorithm.

### Gene set variation analysis (GSVA) and gene ontology (GO) annotation

GSVA analysis and the R package "GSVA" were used to study the variation of biological processes between different TOP2A co-expression patterns^57^. Explicit biological signatures were obtained from the feature marker gene set (downloaded from the MSigDB database v7.1) and the gene set constructed by Maritassan et al. (from the IMvigor210 synbiotic package). GO annotation of TOP2A co-expression-related genes was performed in the R package "Cluster Analyzer" with a cutoff value of FDR < 0.01.

### Adopting ssGSEA immune cell infiltration estimation and TIDE

Single sample gene set enrichment analysis (ssGSEA) to quantify the relative abundance of 28 immune cell types in the tumor microenvironment. The specific signature gene sets used to mark each immune cell type were compiled from a recent study. The relative abundance of each immune cell type was expressed as an enrichment score in the ssGSEA analysis and normalized to a uniform distribution from 0 to 1.

The tumor immune dysfunction and exclusion (TIDE) algorithm proposed by Jiang et al. was used to model different tumor immune evasion mechanisms, including Dysfunction of tumor-infiltrating cytotoxic T lymphocytes (CTLs) and rejection of CTLs by immunosuppressive factors, rejection by immunosuppressive factors^58^.

### Mutation characteristics of significantly mutated genes and tumors

The waterfall feature of the 'R'maf toolkit describes mutations in TOP2A co-expressed genes and smg in the TCGA-LUAD cohort. Mutation signatures extracted from TCGA genomic data were also used in the 'mafools' package. The extracted signature function based on Bayesian variable non-negative matrix decomposition decomposes the mutation portrait matrix into two non-negative matrices 'signature' and 'contribution', where 'signature' represents mutations. The "signature" represents the mutation process and the "contribution" represents the corresponding mutation activity. The signature enrichment function automatically determines the optimal number of extracted mutation signatures and assigns them to each sample based on mutation activity. The extracted CC mutation signatures are compared and annotated with the cancer somatic mutation catalog (universe) by cosine similarity analysis.

### Construction of a prognostic model for TOP2A co-expressed genes

The previous consensus clustering algorithm classified patients into two different TOP2A co-expression patterns. Next, we identified differentially expressed genes (DEG) associated with TOP2A co-expression modifications in different TOP2A co-expression phenotypes. The R package "limma" was used to assess the degradation of LUAD samples between different modification clusters. Specifically, gene expression data were voom normalized and entered into the limit and eBayes functions to calculate differential expression statistics. Deg's significance filtering criteria were set to an adjusted p-value of less than 0.001. We constructed four TOP2A co-expression-related gene scores (TCRGs) using multifactorial Cox regression analysis, which were evaluated according to the following equation the relationship between TOP2A co-expression-related genes and LUAD risk. The risk score formula was: Risk score = ∑(β_i_ ∗ Exp_i_).Clinical information and platform annotation information for the external validation dataset were obtained from the GSE11969 dataset of the Gene Expression Omnibus (GEO) database (http://www.ncbi.nlm.nih.gov/geo), in which a total of 149 LUAD patients were included in the validation dataset of this study.

### Clinical specimens and immunohistochemistry

A total of 141 post-surgical tissue specimens were collected from patients with non-small cell lung cancer at the First Affiliated Hospital of Bengbu Medical College. Patients did not receive radiotherapy or chemotherapy prior to surgery. All specimens were fixed in 4% neutral formaldehyde, paraffin-embedded, and tissue sections were stained to confirm the pathological diagnosis. Clinicopathological staging of lung cancer was determined according to the American Joint Committee on Cancer 8th edition lung cancer staging system. The clinicopathological parameters of the patients are shown in Additional file 1: Table S1.

IHC experimental procedures and scoring criteria for staining results were as previously described^59^. Subsequently, we asked two senior pathologists to score the IHC staining results of lung cancer tissues according to the established scoring criteria. We used PAS-CD31 double staining to show the structure of VM, and we considered the tumor to have the structure of VM when it appeared negative for CD31 and positive for PAS.

### Cell lines and culture, plasmid or lentiviral construction and cell transfections

Human NSCLC cell lines (A549 and H1299) were acquired from the ATCC and were grown in RPMI-1640 media supplemented with 10% FBS at 37 °C and 5% CO_2_.

To down-regulate TOP2A expression, specific small interfering RNAs (si-NC, si-TOP2A) were introduced into non-small cell lung cancer cell lines, and when the cell fusion rate reached 50–60%, cells were transfected with Lipofectamine^®^ 3000 reagent (Thermo Fisher Science) according to the operation manual, thus inhibiting endogenous TOP2A expression. To construct high expression exogenous TOP2A non-small cell lung cancer cell lines using a lentiviral packaging system, the full-length TOP2A gene was cloned into the expression vector pSLenti-EF1-Puro- CMV- 3xFLAG-WPRE (OBioTechnology, Shanghai, China) and introduced into into A549, PC9 and H1299 cells. Infected cells were screened in culture medium containing puromycin (2 μg/ml) for 48 h and then stably passaged in culture medium containing puromycin (1 μg/ml).

### RNA extraction and quantitative real-time polymerase reaction (qRT-PCR)

All experimental procedures for RNA extraction, cDNA synthesis, and quantitative detection of Ct values of target genes were performed as described previously^59^. The final results were normalized to actin (β-actin) expression. The specific primers used are listed in Additional file 2: Table S2. For the analysis of amplification results, relative fold changes were calculated using the 2^−ΔΔCt^ method.

### Western blot assay and staining of the cytoskeleton

Cells were lysed in RIPA buffer (Beyotime, China) and protein concentrations were determined using a BCA kit (Beyotime, China). Equal amounts of proteins were separated on 10% SDS-PAGE and transferred to PVDF membranes. After PVDF membranes were closed with 5% skimmed milk for 1.5 h at room temperature, PVDF membranes were incubated with diluted primary antibody overnight at 4 °C in a shaker. (TOP2A and wnt3a primary antibodies were from Abcam, all other antibodies were from Proteintech.) Some blot strips were cut prior to hybridization with the antibody, and the original blot strips are listed in the supplemental material (Supplementary Original western blot images). The next day, the membranes were washed with TBST three times until clean, and then incubated in freshly prepared specific secondary antibodies for 1.5 h. Subsequently, the membranes were washed again with TBST three times until clean. Finally, the ECL kit (Beyotime, China) was used for detection and developed with Gel Doc 2000 (Bio-Rad).

The cultured NSCLC cytoskeleton was visualized by FITC-ghost pen cyclic peptide immunofluorescence and Comas Brilliant Blue staining. First, 8 × 10^4^ cells per group were inoculated in 6-well plates. On the second day, the apposed cells were fixed with 4% paraformaldehyde at room temperature for 20 min, and then the cell membrane was perforated with 0.2% Triton-X-100 for 5 min, followed by the addition of 1 ml of Kaumas Brilliant Blue R-250 (0.2%) staining solution or FITC-labeled ghost pen cyclopeptide fluorescent staining solution to stain the cytoskeleton. Finally, the cytoskeletal structure was observed with an inverted microscope (IX71, Olympus, Tokyo, Japan).

### CCK8 and colony formation assays

Cells were inoculated overnight in 96-well plates with a cell density of 2000 cells/well and complete culture medium of RPMI-1640. 1.5 h before the assay, each well was replaced with a mixture containing 10 μl CCK-8 reagent and 90 μl complete culture medium. After incubation for 1.5 h at 37 °C under light-proof conditions, the optical density values at 450 nm were measured by enzyme marker.

1000 treated cells in each well of a 6-well plate were cultured for 7–10 days. Subsequently, cells were fixed with 4% paraformaldehyde and stained with 0.1% crystalline violet. After washing and drying, cell colony counting and analysis were performed.

### Cell apoptosis and cell cycle

Apoptotic cells were detected with the Annexin V-FITC/PI Apoptosis Detection Kit (Beyotime, Shanghai, China). Briefly, cells were digested and collected, resuspended with PBS and centrifuged, and finally stained with 5 μl Annexin V-FITC and 10 μl PI for 30 min at room temperature and protected from light. Apoptosis rate of cells was detected by flow cytometry.

Cell cycle analysis was performed using 75% ethanol fixation overnight at 4 °C. After fixation, cells were washed and resuspended with PBS and co-incubated with 10 mg/ml RNase and 1 mg/ml PI for 30 min at 37 °C in the dark. Cell cycle analysis by flow cytometry.

### Tube formation assay and PAS staining of VM networks in vitro

50 μl of thawed matrix gel (Corning, USA) was added to 48-well plates and incubated for 4 h at 37 °C until the matrix gel solidified. A549, PC9 and H1299 cells were suspended in complete culture medium at a density of 5–8 × 10^5^ cells/ml, 200 μl of cell suspension was added to each well and incubated for 12 h. After 12 h, VM structure formation was observed under a microscope (IX73. Olympus) to observe and capture the formation of VM structures. After three-dimensional culture, the tube formation ability of cancer cells was detected by PAS staining.

The specific steps of PAS staining method are as follows: (1) Wash the stromal gel with PBS three times carefully without damaging the tube structure on the stromal gel. (2) Fix with 4% paraformaldehyde for 20 min and carefully wash the matrix gel with PBS again three times. (3) Add 200 μl of periodate to the matrix gel, incubate for 20 min and discard the solution. (4) Add 200 μl Schiff's solution to the matrix gel and incubate for 1 h. Wash carefully with PBS three times. (5) Observe and capture the formation of VM structures under a microscope (IX73, Olympus).

### Statistical analysis

Statistical analysis of all experimental data was performed using GraphPad Prism 9.0. The *t*-test was used for comparison between two groups, and one-way ANOVA was used for comparison between multiple groups. A Chi-square test was performed for clinicopathological characteristics of patients positive for TOP2A and Wnt3a expression. Kaplan–Meier curves and log-rank tests were used to compare survival outcomes across subgroups. The independent prognostic value of OS clinical characteristics was assessed using univariate Cox proportional risk regression analysis. The prognostic ability of OS prediction models was assessed using subject operating characteristic (ROC) curves (R package "timeROC") and area under the curve (AUC) values. All statistical tests were two-tailed, and p < 0.05 was considered statistically significant.

### Ethics approval and consent to participate

Written informed consent was obtained from all patients and approved by the Ethics Committee of Bengbu Medical College (No. 2020KY035), and the study was conducted according to the ethical guidelines of the Declaration of Helsinki.

## Supplementary Information

Supplementary Figure 1.

Supplementary Figure 2.

Supplementary Figure 3.

Supplementary Legends.

Supplementary Information.

Supplementary Table S1.

Supplementary Table S2.

Supplementary Table S3.

Supplementary Table S4.

## Data Availability

Publicly available datasets which can be found in the cancer genome atlas (TCGA) database or gene expression omnibus (GEO) were used in this study.
