# Supplementary figures and images for: The role of TOP2A in immunotherapy and vasculogenic mimicry in non-small cell lung cancer and its potential mechanism
Source: Sci Rep. 2023 Jul 5;13:10906. doi: 10.1038/s41598-023-38117-6 (PMC10322841; doi:10.1038/s41598-023-38117-6)

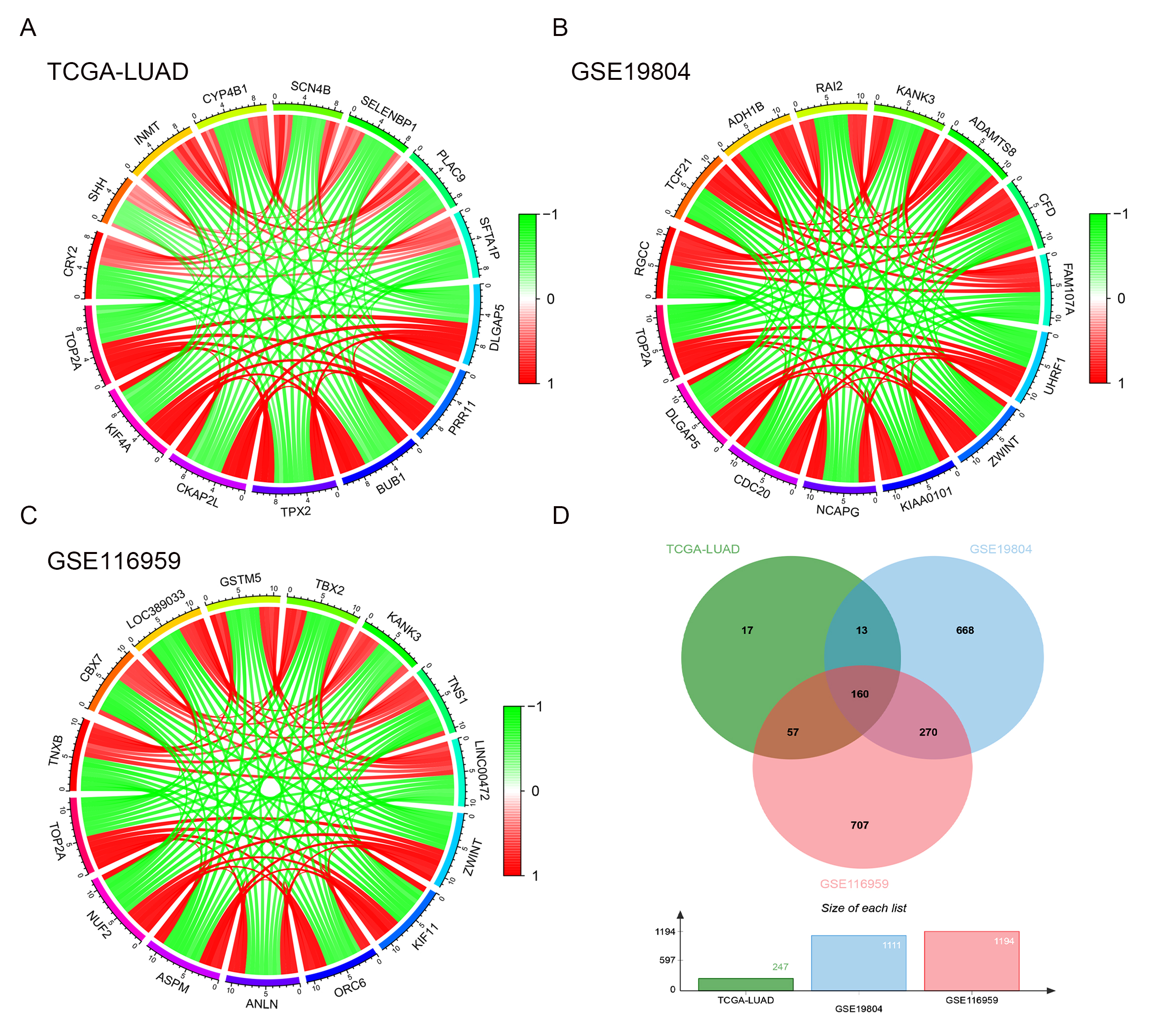

Supplement: Supplementary file 1 — Supplementary Figure 1. [file 41598_2023_38117_MOESM1_ESM.jpg]

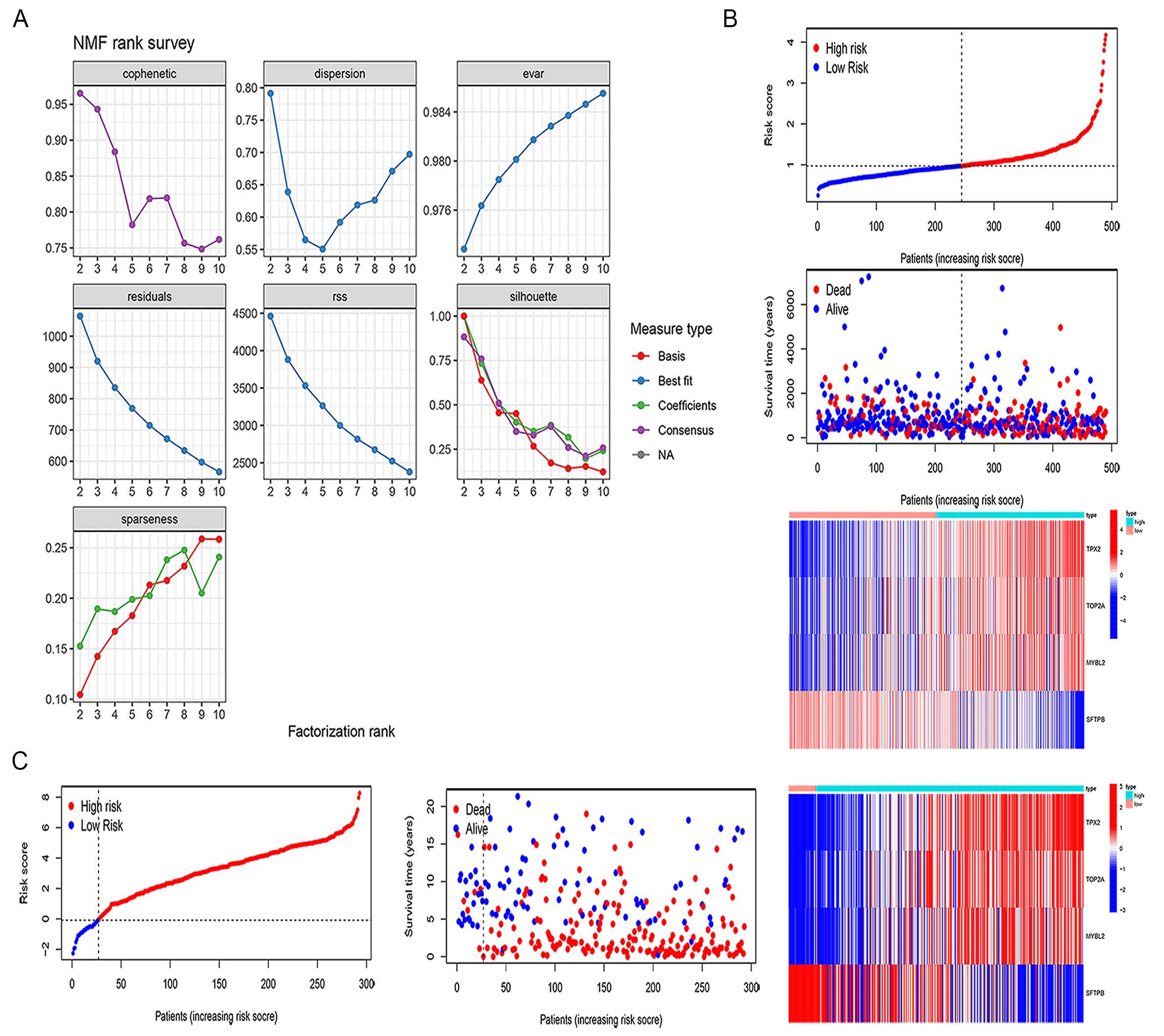

Supplement: Supplementary file 2 — Supplementary Figure 2. [file 41598_2023_38117_MOESM2_ESM.jpg]

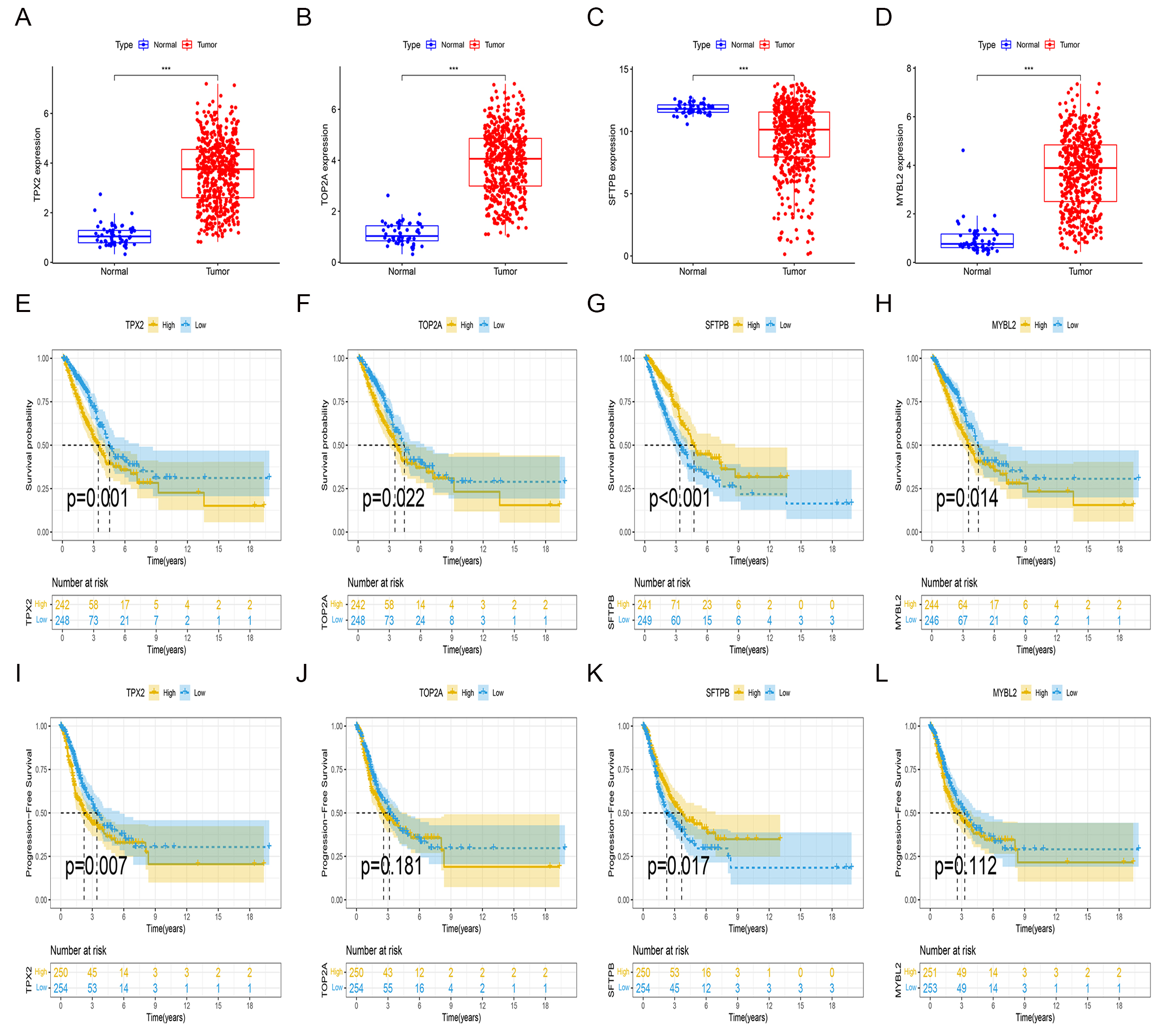

Supplement: Supplementary file 3 — Supplementary Figure 3. [file 41598_2023_38117_MOESM3_ESM.jpg]
