# Supplementary material for: The role of TOP2A in immunotherapy and vasculogenic mimicry in non-small cell lung cancer and its potential mechanism
Source: Sci Rep. 2023 Jul 5;13:10906. doi: 10.1038/s41598-023-38117-6 (PMC10322841; doi:10.1038/s41598-023-38117-6)
Supplement: Supplementary file 4 — Supplementary Legends. [file 41598_2023_38117_MOESM4_ESM.docx]

**The role of TOP2A in immunotherapy and Vasculogenic mimicry in non-small cell lung cancer and its potential mechanism**

Jiatao Wu^1,3a^, Lei Zhang^2a^, Wenjuan Li^1,3a^, Luyao Wang^3^, Qianhao Jia^3^, Fan Shi^3^, Kairui Li^3^, Lingli Liao^5^, Yuqi Shi^3^&Shiwu Wu ^3,4🖂^

^1^Anhui Province Key Laboratory of Clinical and Preclinical Research in Respiratory Disease，Molecular Diagnosis Center，First Affiliated Hospital，Bengbu Medical College, 287 Changhuai Road, Bengbu 233004, Anhui, China.

^2^Department of Oncology Surgery, the Second Affiliated Hospital of Bengbu Medical College, Anhui Province, 233080, China.

^3^ Department of Pathology, The First Affiliated Hospital of Bengbu Medical College, Bengbu, People’s Republic of China.

^4^ Department of Pathology, Bengbu Medical College, Anhui Province, 233030, China.

^5^Department of Radiation Oncology, the First Affiliated Hospital of Bengbu Medical College, Anhui Province, 233000, China.

a: Author contributed equally

Correspondence should be addressed to Shiwu Wu; wushiwu@bbmc.edu.cn.

*Corresponding authors:

**Shiwu Wu:** Department of Pathology, Bengbu Medical College, Anhui Province, 233030, China; Department of Pathology, The First Affiliated Hospital of Bengbu Medical College, Bengbu, People’s Republic of China.

**Supplementary Table S1:** Patient clinicopathological features.

**Supplementary Table S2:** Genes significantly co-expressed with TOP2A in TCGA-LUAD, GSE19804, GSE116959 dataset；Gene intersection of three study cohorts； Differential genes between C1 and C2；17 LUAD prognosis-related genes.

**Supplementary Table S3:** The correlation between VM and clinicopathological characteristics in NSCLC.

**Supplementary Table S4:** Correlation of TOP2A expression pattern and tumor cytoskeleton and motility.

**Supplementary Original western blot images:** Original western blot images of Figure4 B; Figure7 F; Figure7 G; Figure8 F in the manuscript.

**Supplementary Figure S1**

Genes significantly co-expressed with TOP2A from the lung cancer dataset. (A-C) The top 14 genes significantly co-expressed with TOP2A in the three study cohorts. (A) TCGA-LUAD; (B) GSE19804; (C) GSE116959; (D) Venn diagram of the three study cohorts.

**Supplementary Figure S2**

Construction and validation of the TOP2A co-expression-associated gene risk model by multifactorial Cox regression analysis. (A) Factorization rank of k=2-10. (B-C) Distribution of risk curves and number of patients with TOP2A co-expression-associated gene risk scores; (B) internal test set（TCGA-LUAD）; (C) external validation set（GSE11969）.

**Supplementary Figure S3**

Expression and prognosis of TOP2A, TPX2, MYBL23 and SFTPB in LUAD in TOP2A co-expression-related gene risk model. (A-D) Expression of TOP2A, TPX2, MYBL23 and SFTPB in lung cancer and normal lung tissues. (E-H) Overall survival (OS) of high and low expression cohorts of TOP2A, TPX2, MYBL23 and SFTPB. (I-L) Progression-free survival (PFS) in the TOP2A, TPX2, MYBL23 and SFTPB high and low expression cohorts.
