# Supplementary material for: The role of TOP2A in immunotherapy and vasculogenic mimicry in non-small cell lung cancer and its potential mechanism
Source: Sci Rep. 2023 Jul 5;13:10906. doi: 10.1038/s41598-023-38117-6 (PMC10322841; doi:10.1038/s41598-023-38117-6)
Supplement: Supplementary file 5 — Supplementary Information. [file 41598_2023_38117_MOESM5_ESM.pdf]

Figure4 B

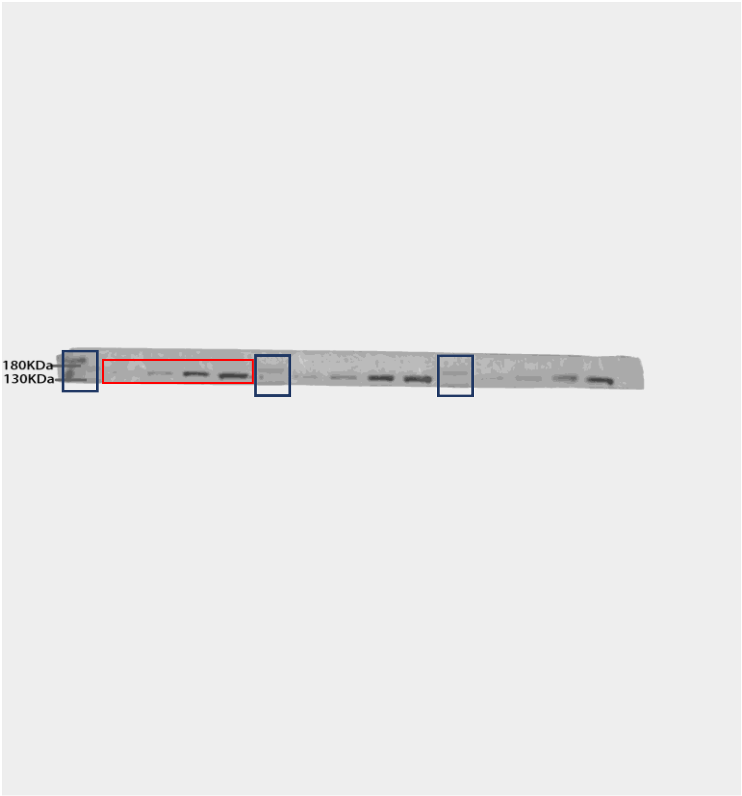

Original western blot image of TOP2A-OE.

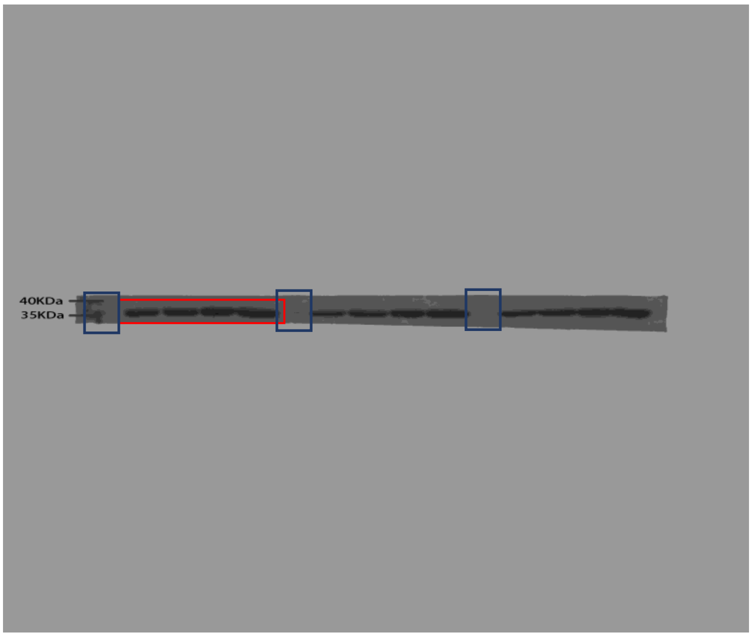

Original western blot image of GAPDH (TOP2A-OE).

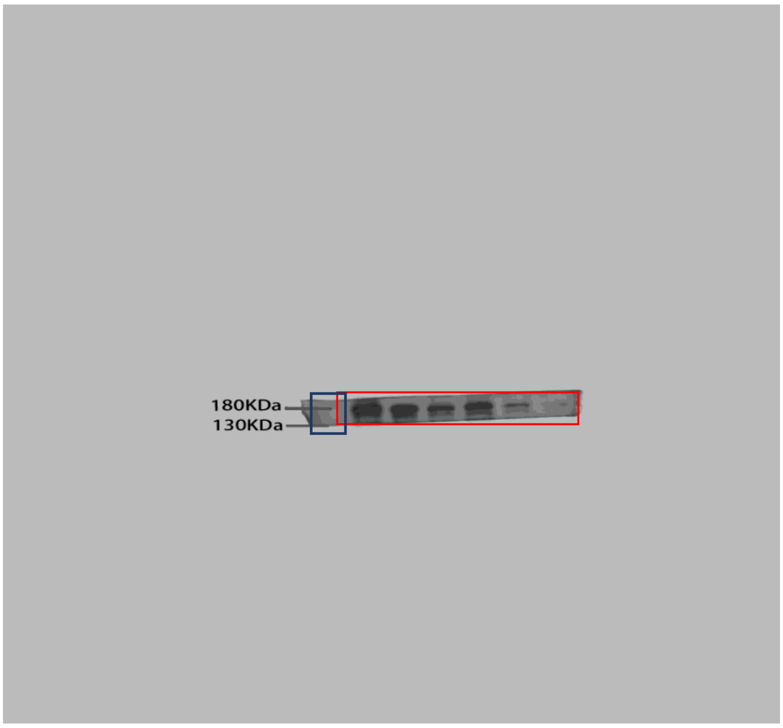

Original western blot images of TOP2A-Sirna, this band was cut in half for the other proteins before hybridization with the antibody.

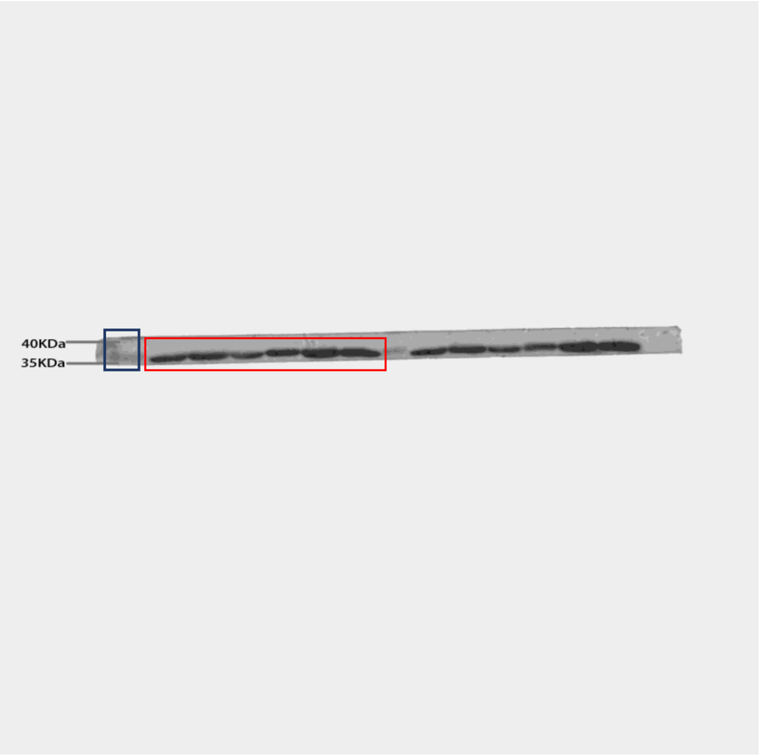

Original western blot image of GAPDH (TOP2A-Sirna).

Figure7 F

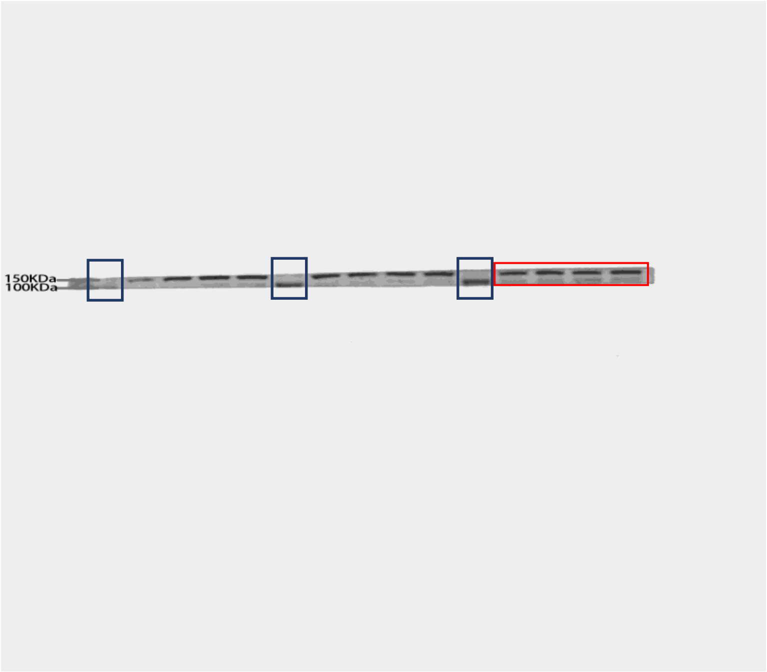

Original western blot image of ZEB1.

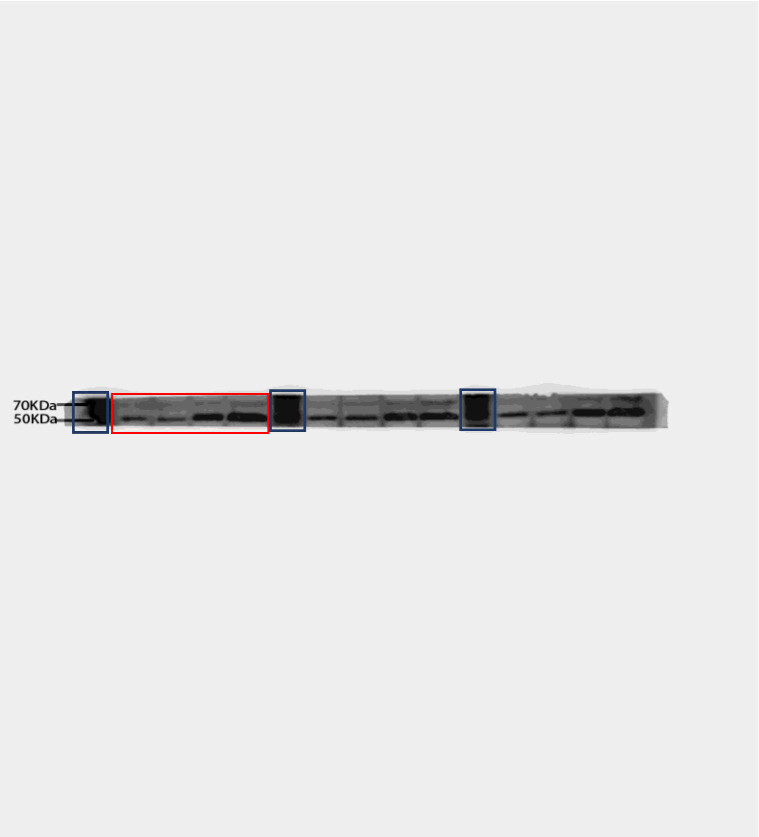

Original western blot image of PD-L1.

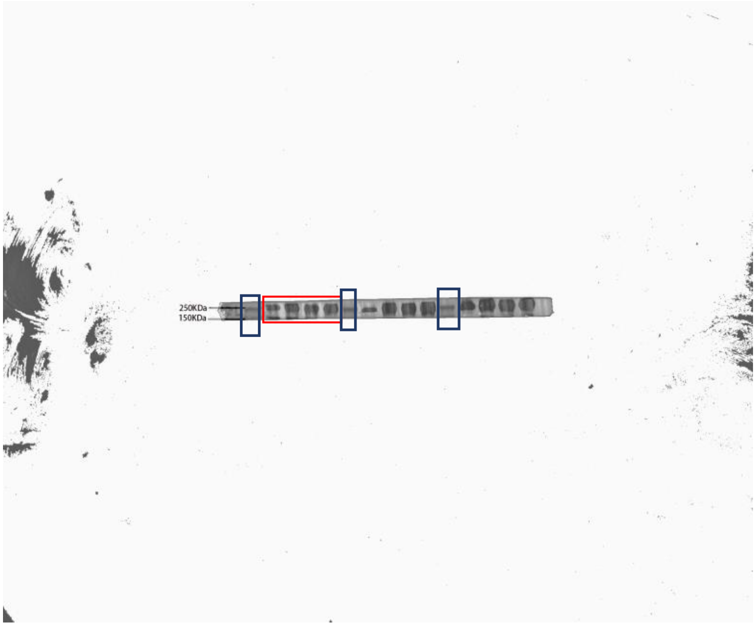

Original western blot image of Laminin.

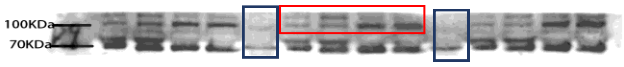

Original western blot image of MMP9.

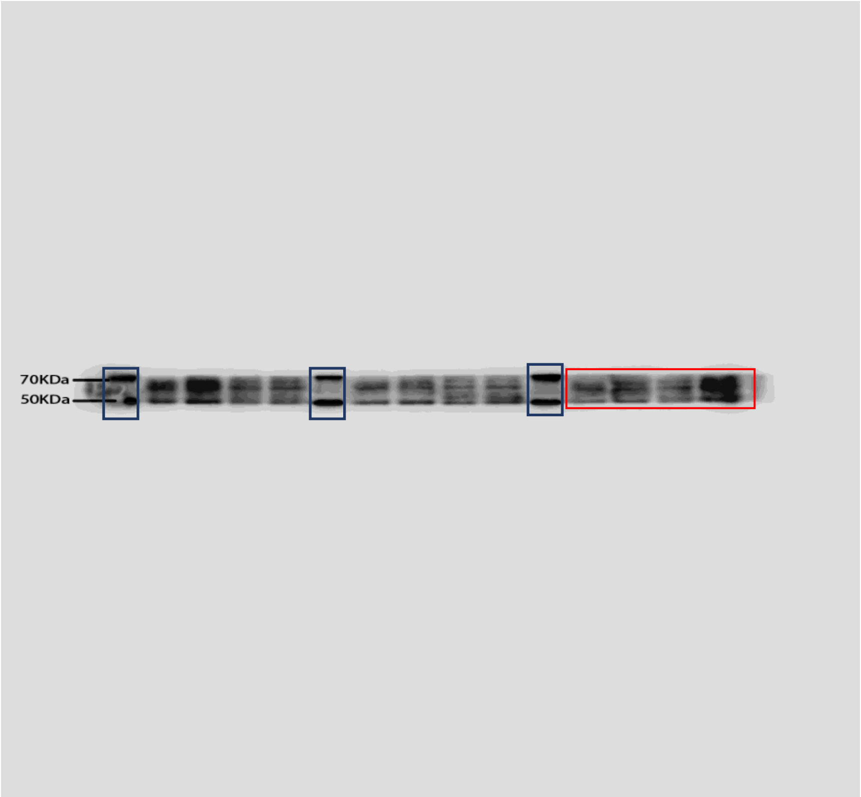

Original western blot image of MMP2.

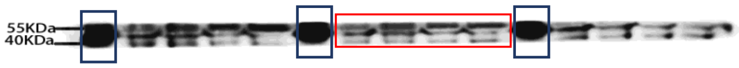

Original western blot image of VEGFA.

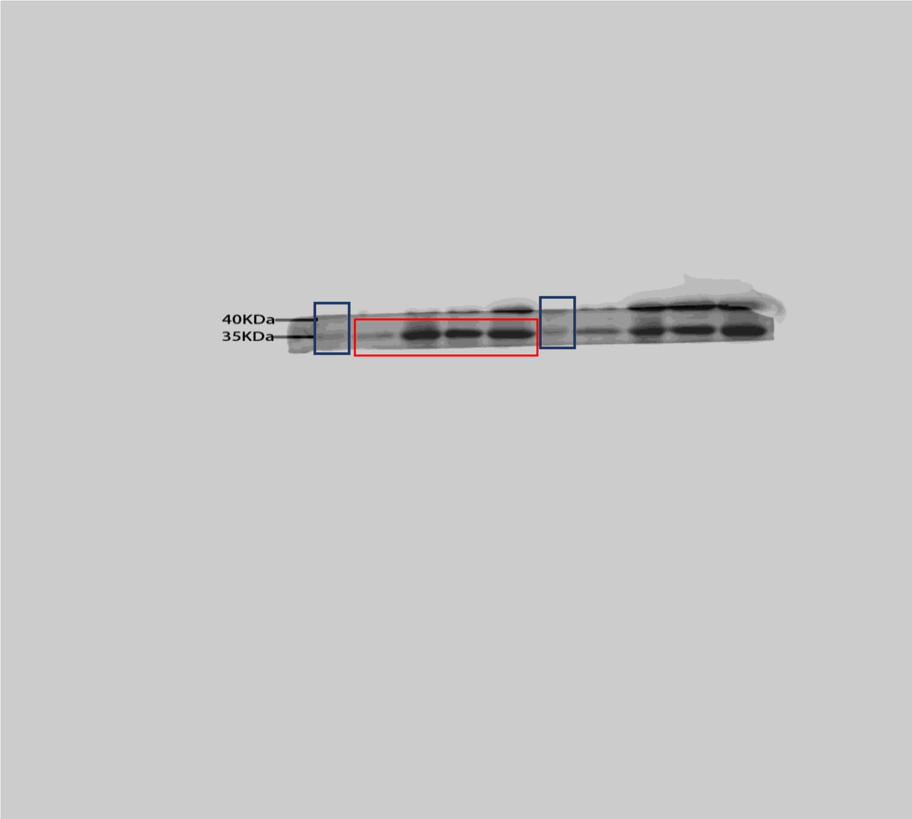

Original western blot images of Wnt3a; this band was partially cleaved for the detection of other proteins before hybridization with the antibody.

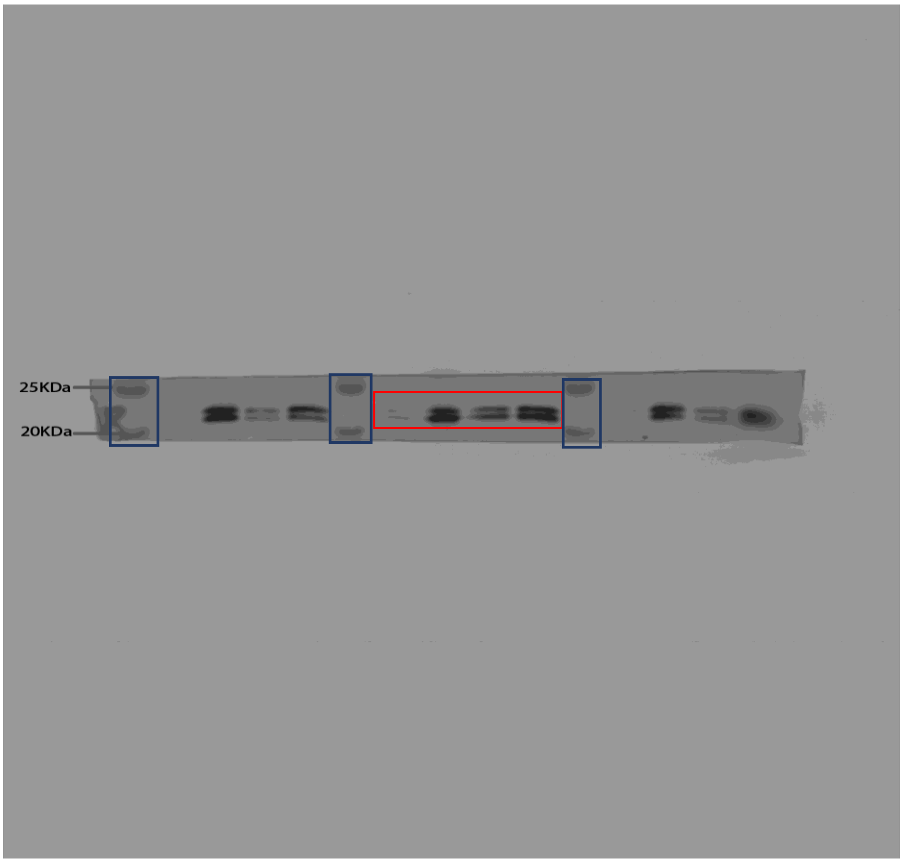

Original western blot image of RHOA.

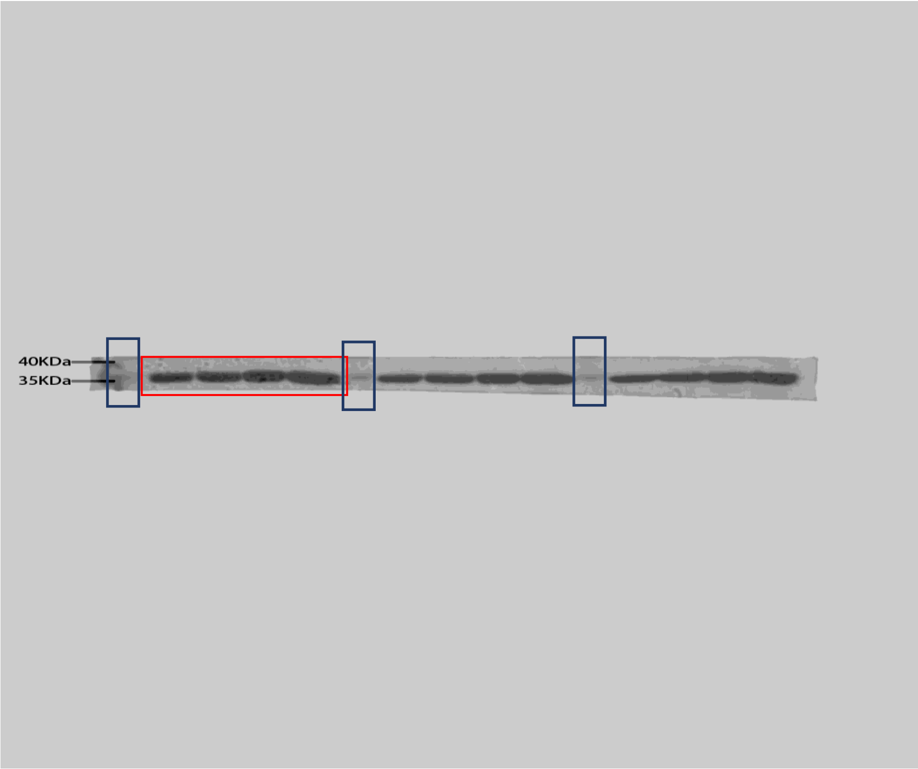

Original western blot image of GAPDH.

Figure7 G

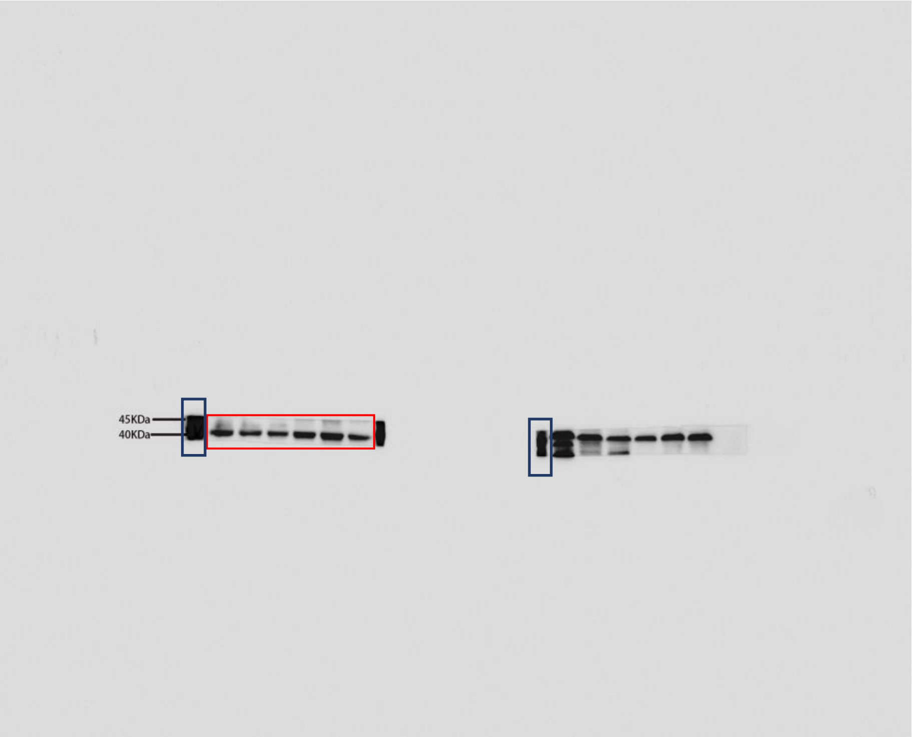

Original western blot image of VEGFA.

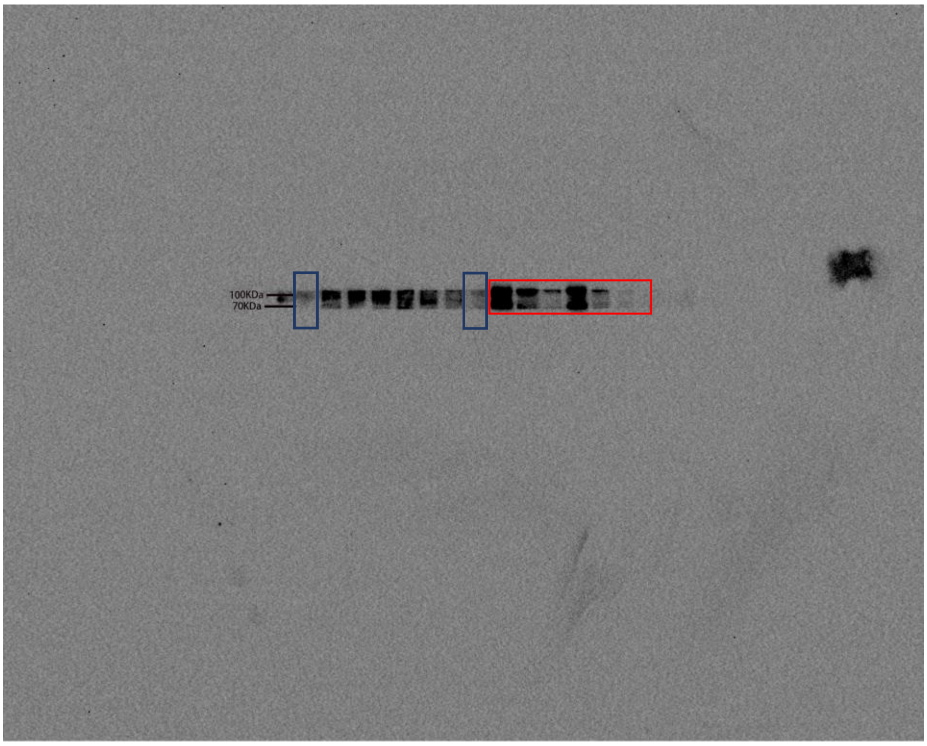

Original western blot image of MMP9.

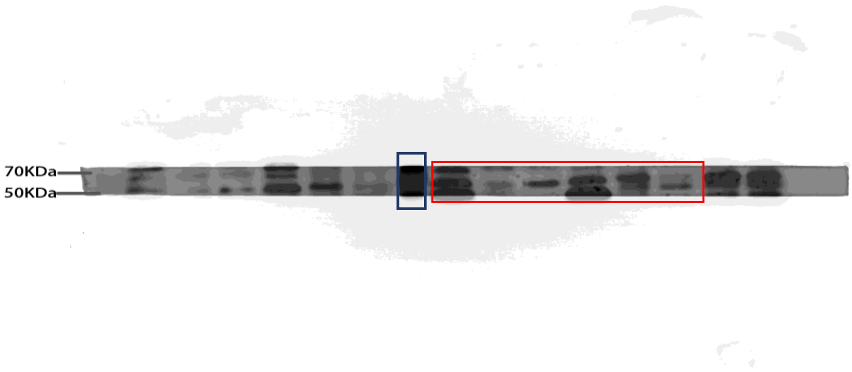

Original western blot image of MMP2.

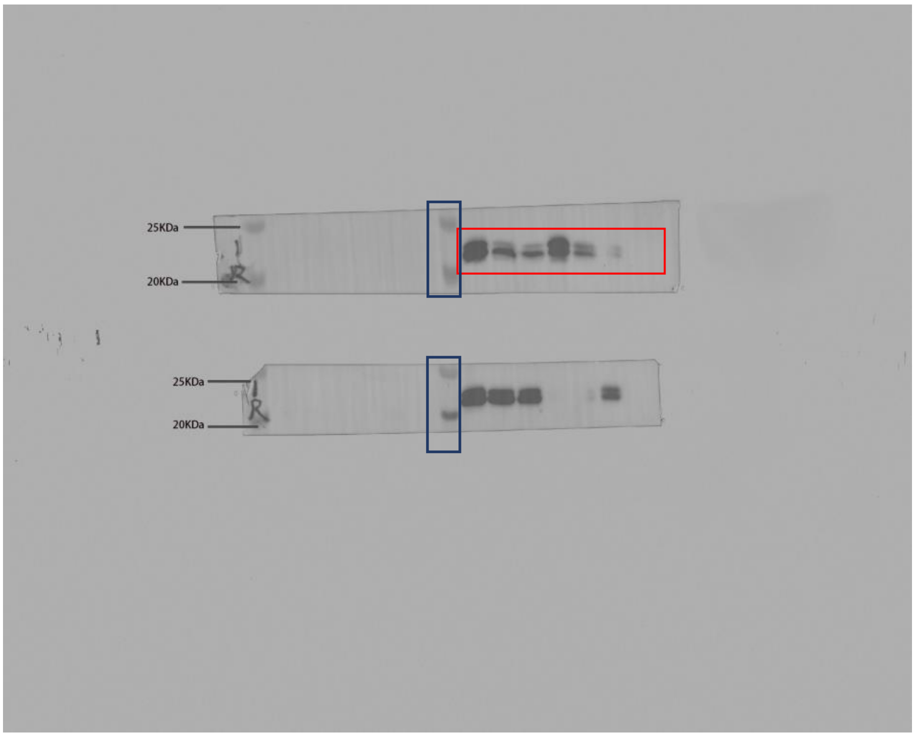

Original western blot images of RHOA; this band was partially cleaved before hybridization with the antibody for detection of other proteins.

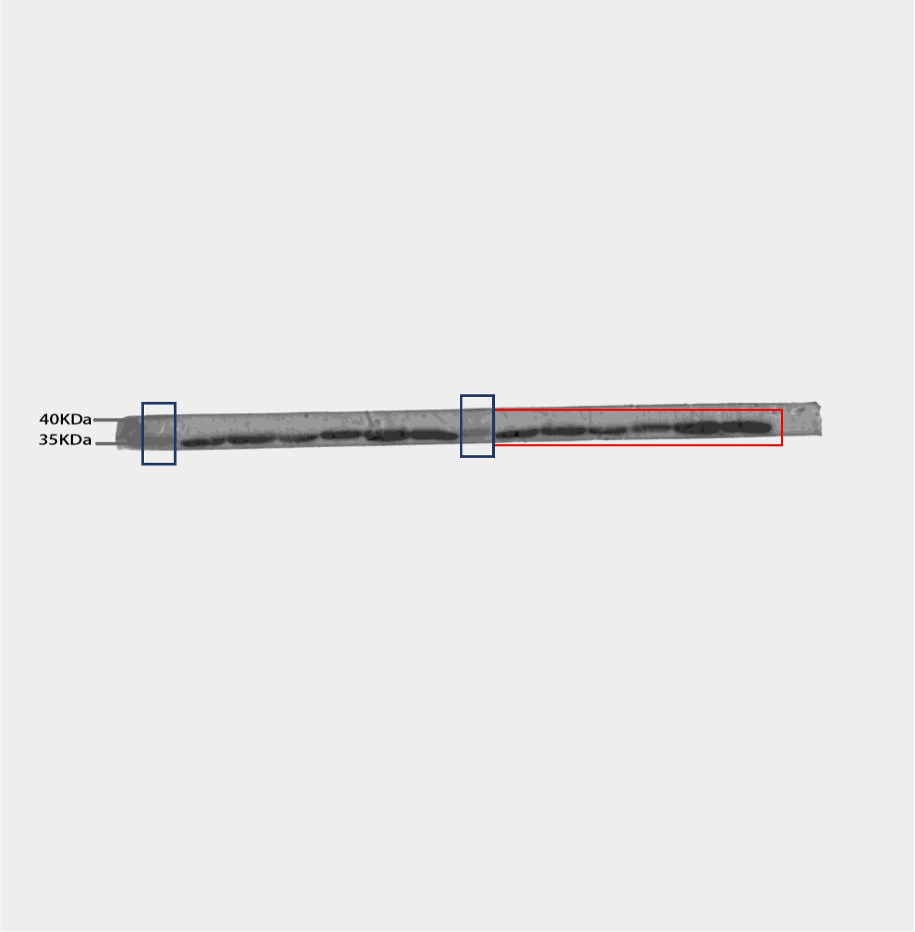

Original western blot image of GAPDH.

Figure8 F

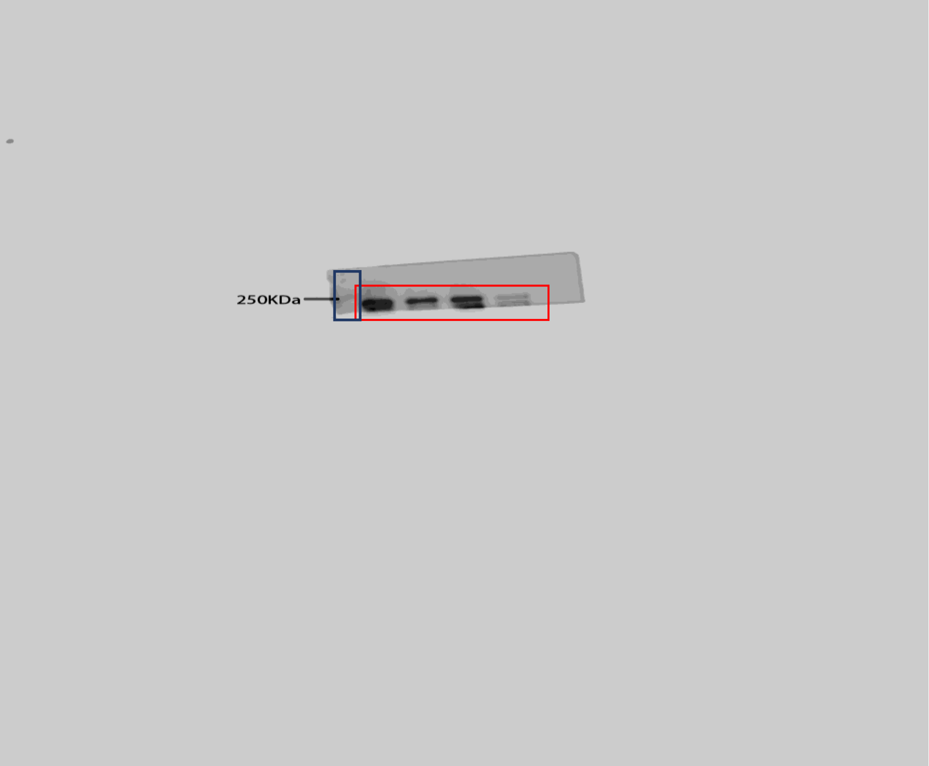

Original western blot images of Laminin; this band was partially cleaved for the detection of other proteins before hybridization with the antibody.

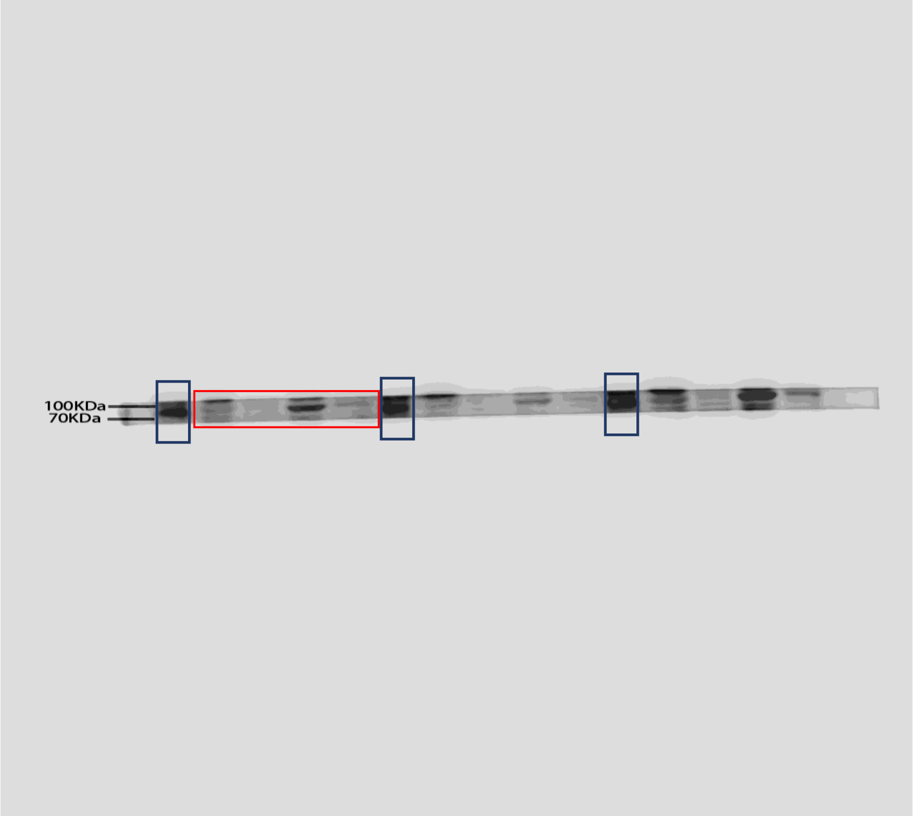

Original western blot image of MMP9.

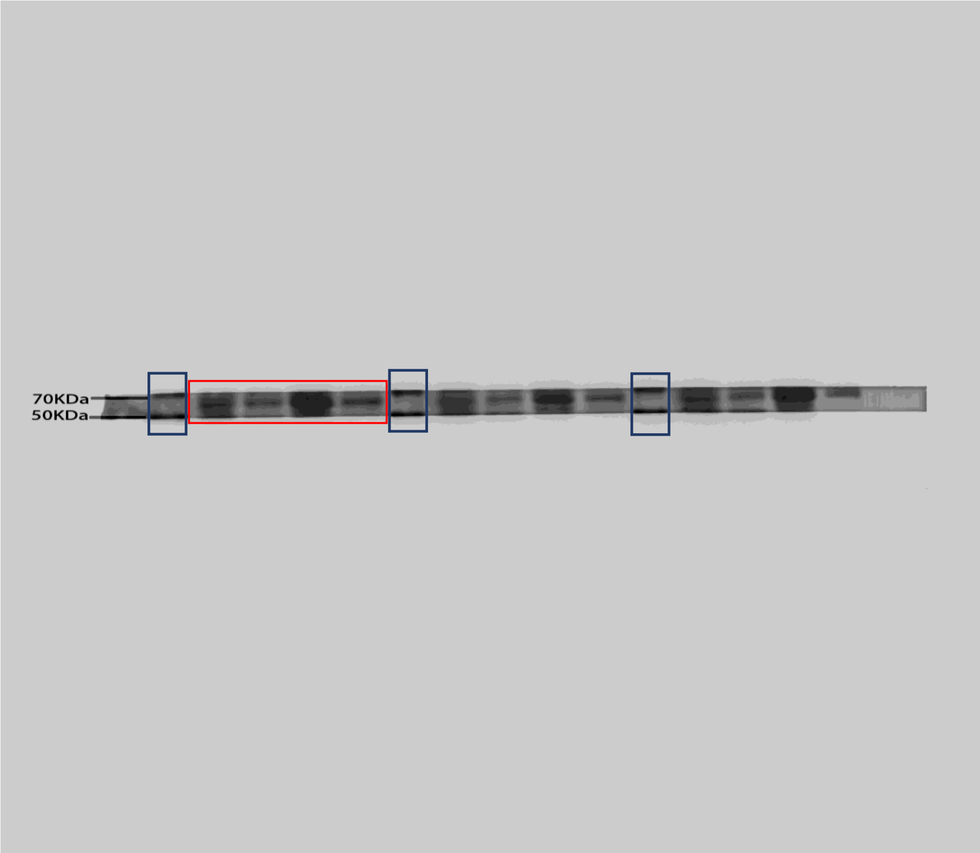

Original western blot image of MMP2.

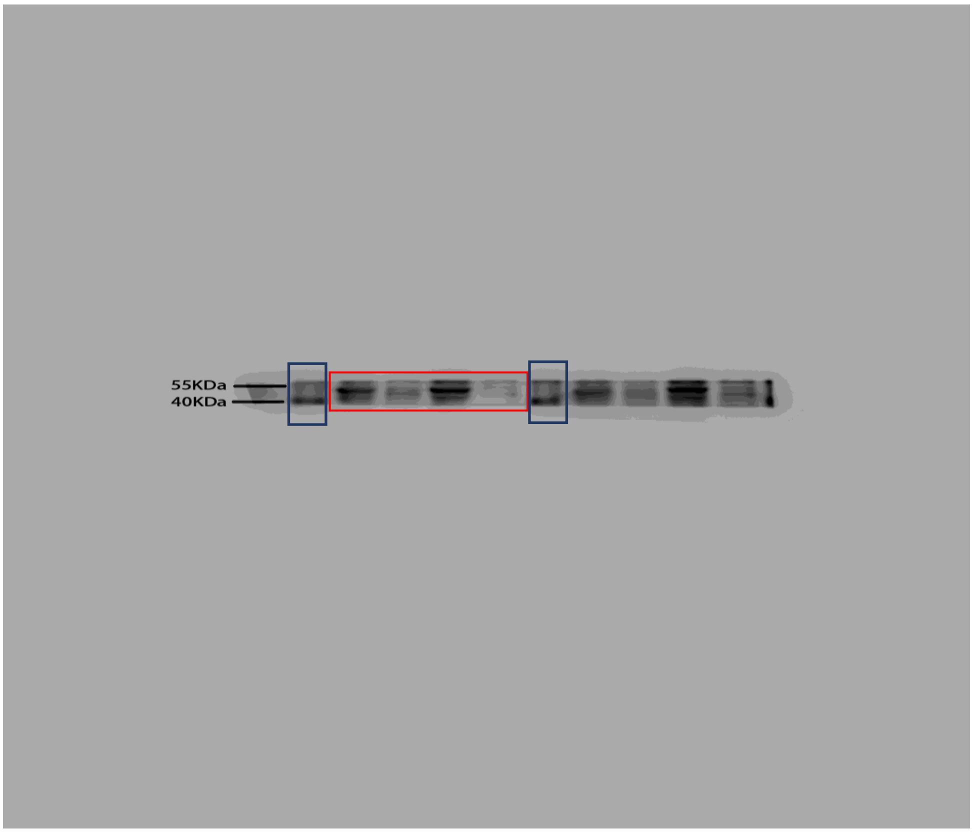

Original western blot images of VEGFA; this band was partially cleaved for the detection of other proteins prior to hybridization with the antibody.

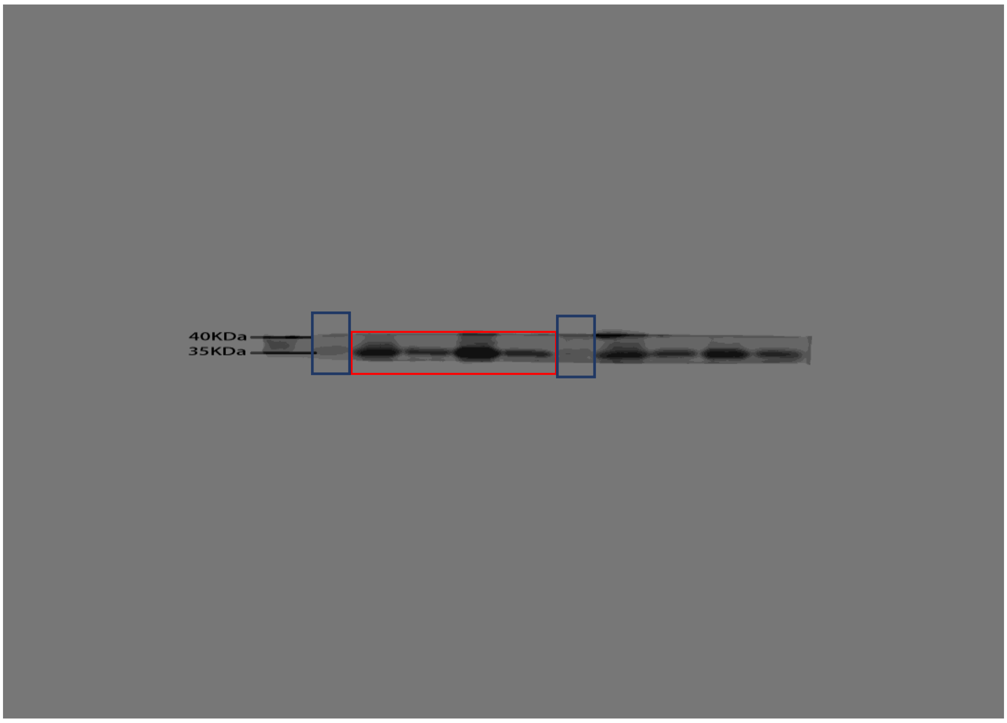

Original western blot images of Wnt3a; this band was partially cleaved for detection of other proteins prior to hybridization with the antibody.

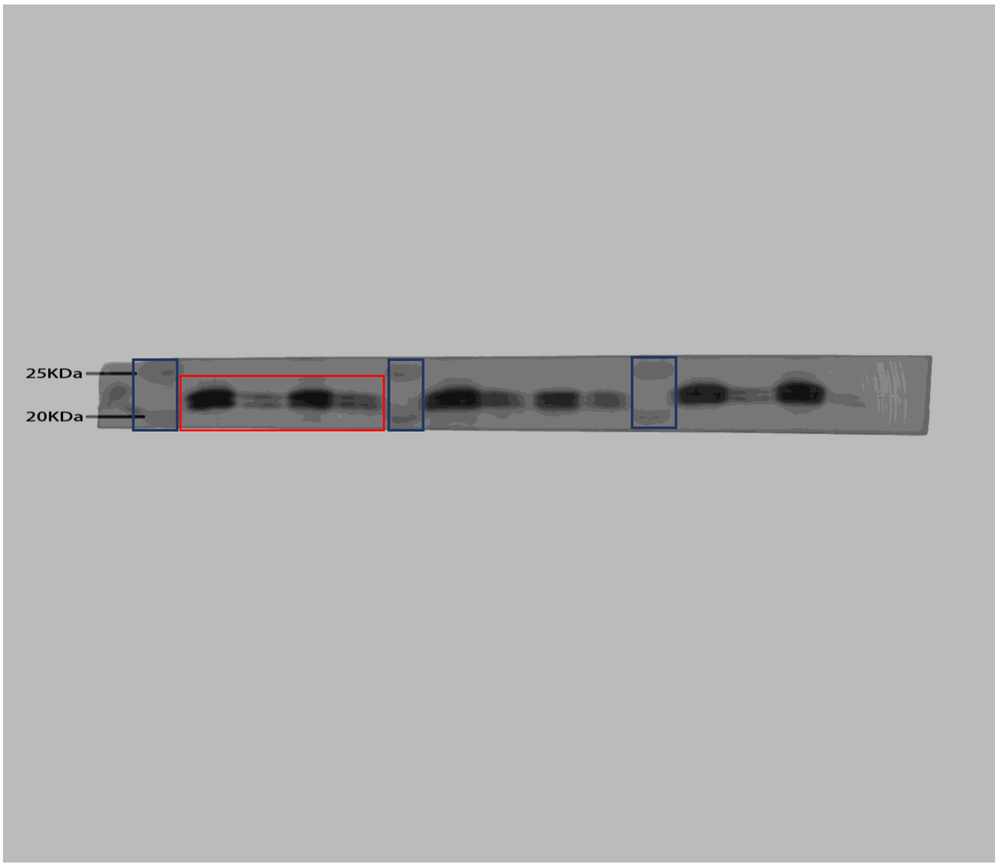

Original western blot image of RHOA.

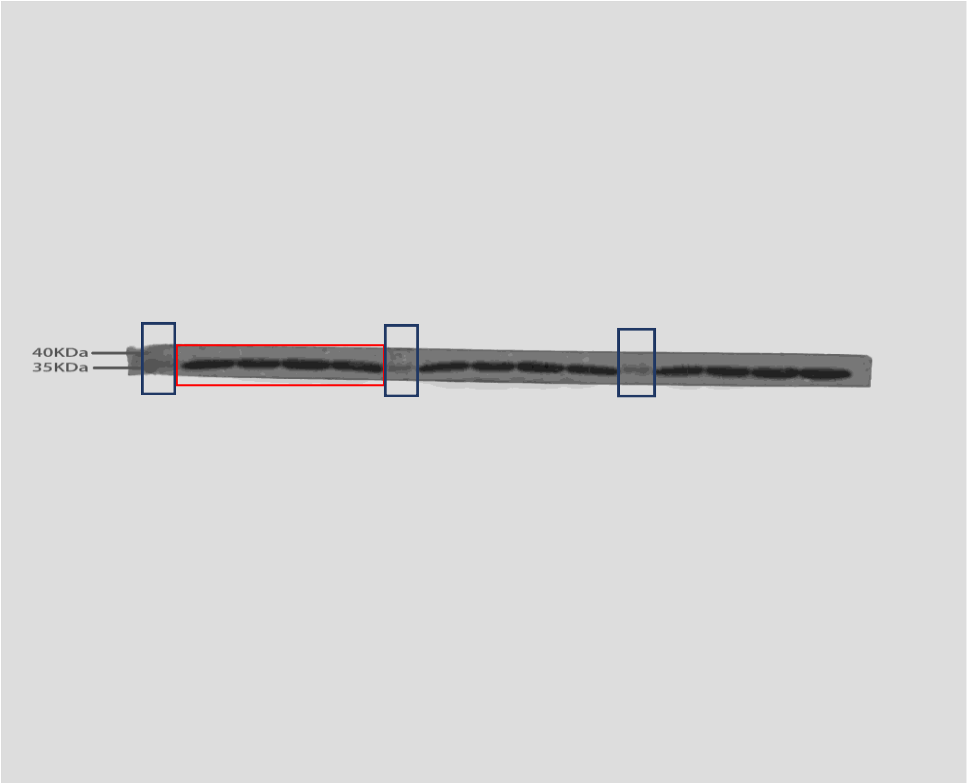

Original western blot image of GAPDH.

Note: the red box indicates the area of the original blot used in the main image; the blue box indicates the hole where the protein mark is located. Most of the original strips provided were replicated three times in a single experiment.
