# Supplementary material for: The role of TOP2A in immunotherapy and vasculogenic mimicry in non-small cell lung cancer and its potential mechanism
Source: Sci Rep. 2023 Jul 5;13:10906. doi: 10.1038/s41598-023-38117-6 (PMC10322841; doi:10.1038/s41598-023-38117-6)
Supplement: Supplementary file 6 — Supplementary Table S1. [file 41598_2023_38117_MOESM6_ESM.pdf]

Table S1: Patient clinicopathological features

Patients characteristics	Frequency (n)	Percentage (%)
Age (years)		
＜60	65	46.1
⩾60	76	53.9
Gender		
Female	61	43.3
Male	80	56.7
Smoking		
No	93	66
Yes	48	34
Gross Type		
Central	72	51.1
Peripheral	69	48.9
Histologic Type		
SCC	21	14.9
Ade	120	85.1
Grade		
Well	30	21.3
Moderate	81	57.4
Poor	30	21.3
LNM		
No	80	56.7
Yes	61	43.3
TNM stage		
Ⅰ	61	43.3
Ⅱ	35	24.8
Ⅲ	45	31.9
