# Supplementary material for: The role of TOP2A in immunotherapy and vasculogenic mimicry in non-small cell lung cancer and its potential mechanism
Source: Sci Rep. 2023 Jul 5;13:10906. doi: 10.1038/s41598-023-38117-6 (PMC10322841; doi:10.1038/s41598-023-38117-6)
Supplement: Supplementary file 7 — Supplementary Table S2. [file 41598_2023_38117_MOESM7_ESM.pdf]

Supplementary Table S2: Genes significantly co-expressed with TOP2A in TCGA-LUAD, GSE19804, GSE116959 dataset； Gene intersection of three study cohorts； Differential genes between C1 and C2；17 LUAD prognosis-related genes.

TCGA-LUAD	GSE19804	GSE116959	Gene intersection	Differential genes between C1 and C2	17 LUAD prognosis-related genes
C1orf112	A2M	AARSD1	C1orf112	TPX2	TPX2
DBF4	ABCA6	ABCA6	NCAPD2	TOP2A	TOP2A
E2F2	ABCA8	ABCA8	ANLN	MYBL2	MYBL2
NCAPD2	ABCA9	ABCE1	BRCA1	UBE2C	UBE2C
ANLN	ABCB6	ABCF2	TACC3	C16orf89	C16orf89
BRCA1	ABI3BP	ABI3BP	DEPDC1	SFTA1P	SFTA1P
TACC3	ABLIM1	ABTB1	DEPDC1B	SUSD2	SUSD2
POLA2	ABLIM3	ACACB	R3HDM1	NAPSA	NAPSA
DEPDC1	ACACB	ACADL	RAD51	FOLR1	FOLR1
DEPDC1B	ACADL	ACLY	MCM10	SFTA2	PEBP4
SPDL1	ACKR1	ACTL6A	ASPM	PEBP4	GGTLC1
R3HDM1	ACTN2	ADAMTS8	TRIP13	GGTLC1	CYP4B1
RAD51	ACVRL1	ADCY4	HMMR	CYP4B1	SCGB3A1
NDC1	ADAM12	ADH1A	MCM2	SCGB3A1	SFTPD
MCM10	ADAMTS15	ADH1B	GTSE1	SCGB3A2	SFTPB
MTHFD2	ADAMTS8	ADH1C	MCM6	SFTPD	PGC
ASPM	ADAMTS9-AS2	ADRB2	SPAG5	SFTPB	SFTPC
PRR11	ADAMTSL3	ADSL	UBE2T	CTSE	
TRIP13	ADARB1	AGER	NDC80	PIGR	
HMMR	ADCY4	AGMAT	ORC1	PGC	
MCM2	ADH1B	AGRP	RAD54L	SFTPA1	
GTSE1	ADHFE1	AHCY	AURKA	SFTPA2	
WDR62	ADIRF	AIMP2	TPX2	FGB	
MCM6	ADRB1	AK1	BIRC5	SFTPC	
UNG	ADRB2	AKAP13	KIF4A	SCGB1A1	
SPAG5	AF131215.8	ALDH18A1	ORC6		
UBE2T	AFF3	ALDH2	CLSPN		
RBL1	AGER	ALG10	CDC45		
NDC80	AGTPBP1	ALG3	CDC6		
ORC1	AGTR1	ALOX5	CENPM		

RAD54L	AHCY	AMICA1	POLE2
AURKA	AIMP2	ANAPC11	CDKN3
TPX2	AKAP12	ANKHD1-EIF4EBP3	GINS1
BIRC5	AKR1A1	ANKLE2	MYBL2
KIF4A	AKT3	ANLN	E2F1
ORC6	ALDH18A1	ANP32E	CENPI
WDR76	ALDH2	AOC3	OIP5
CLSPN	ALDOA	ARHGAP11A	MCM4
CDC45	ALG1	ARHGAP11B	ASF1B
CDC6	ALG3	ARHGAP31	CCNE1
MSH2	ALG8	ARHGEF17	EZH2
CDC7	ALS2CL	ARL5B	NCAPG
CENPM	AMOTL1	ARMC10	FOXM1
POLE2	AMOTL2	ASAP3	RAD51AP1
CDKN3	ANAPC11	ASF1B	TIMELESS
VRK1	ANGPT1	ASPM	CDCA3
GINS1	ANGPTL1	ATAD2	GMNN
MYBL2	ANKRD22	ATAD5	TTK
E2F1	ANKRD29	ATIC	KIF20A
FAM83D	ANKRD44	ATP1A2	LMNB1
CENPI	ANKS1A	ATP1B2	ECT2
OIP5	ANLN	ATP5G1	CENPA
POP1	ANO6	AURKA	CDC20
MCM4	ANXA11	AURKB	NEK2
ASF1B	AOC3	AVL9	CENPF
CCNE1	AOX1	B3GNT1	KIF14
EZH2	APEX1	B3GNT4	HELLS
UBE2S	APOA1BP	B4GALT2	CENPL
KPNB1	AQP1	BAIAP2L1	MND1
PSMD11	AQP4	BARD1	ZWINT
EFTUD2	ARGLU1	BCCIP	CENPK
NEIL3	ARHGAP21	BCL2L12	STIL
NCAPG	ARHGAP29	BCO2	HJURP
FOXM1	ARHGAP31	BEND7	MCM8
RAD51AP1	ARHGAP44	BIRC5	DLGAP5
RFC5	ARHGAP6	BLM	PAICS
TIMELESS	ARHGEF19	BMS1P4	PPAT
CDCA3	ARHGEF26	BOLA3	E2F8
FBXO5	ARHGEF3	BOP1	FAM64A
GMNN	ARHGEF39	BORA	GINS2
TTK	ARHGEF6	BRCA1	FIGNL1
KIF20A	ARRB1	BRCA2	CCNB1
LMNB1	ART4	BRIP1	CDCA8

TARS	ASF1B	BRIX1	TROAP
NUP155	ASPA	BTG2	ESPL1
ECT2	ASPM	BTNL9	CKAP2
CENPA	ATAD2	BUB1	BORA
MSH6	ATAD5	BUB1B	DSCC1
CDC20	ATIC	BUB3	TCF19
STMN1	ATOH8	BYSL	NUSAP1
NEK2	ATP10B	BZW1	KIF23
CENPF	ATP1A2	BZW2	CENPO
KIF14	ATP5G1	C10orf116	KIF11
HELLS	AUNIP	C10orf2	CEP55
CENPL	AURKA	C10orf67	DNA2
MASTL	AURKB	C11orf24	BARD1
TMPO	AVL9	C11orf48	CENPE
NCAPH	AX748273	C11orf80	FANCI
MND1	B3GNT3	C11orf82	PLK4
KIF18A	B4GALT2	C11orf83	KIF2C
ZWINT	B4GALT7	C12orf32	NUF2
CENPK	BAI3	C12orf48	DTL
CDK2	BARD1	C12orf66	FANCD2
STIL	BC022047	C12orf73	CCNA2
HJURP	BCHE	C13orf15	CDCA5
CSE1L	BCL2L12	C13orf27	NCAPG2
C17orf53	BCL2L15	C14orf132	GINS4
MCM8	BCL9	C14orf33	MKI67
DLGAP5	BDH1	C15orf23	CHEK1
PKMYT1	BDNF	C15orf42	NCAPD3
PAICS	BIRC5	C16orf59	SPC25
PPAT	BMP2	C16orf88	SUV39H2
KNSTRN	BMPER	C17orf58	CENPH
E2F8	BNIP2	C17orf75	SKA1
FAM64A	BOLA3	C17orf96	ATAD2
DKC1	BORA	C18orf56	BUB1B
NOL11	BPHL	C19orf40	CCNB2
GINS2	BRAT1	C19orf48	CDC25C
PSMC3IP	BRCA1	C19orf59	MIS18A
FIGNL1	BRCA2	C1QTNF7	CHAF1B
RFC3	BRIP1	C1orf112	RECQL4
CCNB1	BTG1	C1orf135	RACGAP1
PSRC1	BTNL9	C1orf186	SPC24
CDCA8	BUB1	C1orf74	C16orf59
TROAP	BUB1B	C20orf194	CCNF
ESPL1	BZW2	C20orf24	SGOL2
CKAP2	C10orf128	C2orf29	KIF15

BORA	C10orf35	C2orf40	RFC4
SMC2	C10orf54	C2orf47	CDC25A
CTSV	C11orf24	C3orf14	POC1A
DSCC1	C11orf80	C3orf26	MAD2L1
TCF19	C12orf45	C4orf21	PTTG1
NUSAP1	C12orf49	C4orf48	MELK
KIF23	C14orf132	C5orf22	SKA3
CENPO	C14orf166	C5orf28	LRR1
KIF11	C14orf169	C5orf34	CENPN
CEP55	C14orf2	C6orf225	KIAA0101
KIF20B	C16orf13	C7orf11	RRM1
DNA2	C16orf59	C7orf44	CDT1
BARD1	C19orf40	C7orf70	CHAF1A
CENPE	C1QTNF2	C8orf51	TK1
GAS2L3	C1QTNF6	C8orf76	PBK
DENR	C1QTNF7	C9orf100	FEN1
FANCI	C1orf112	C9orf140	CKAP2L
PLK4	C1orf21	C9orf174	BUB1
KIF2C	C1orf74	CA4	CDK1
NUF2	C20orf194	CACNA2D2	CDCA4
DTL	C2orf40	CAD	SHCBP1
FANCD2	C4orf48	CALCOCO1	RRM2
CCNA2	C5orf34	CALCRL	C5orf34
SKP2	C5orf46	CAPN3	CKS1B
PRIM2	C7	CARD16	EXO1
MTFR2	C8orf88	CARS	UBE2C
CDCA5	CA3	CASC5	CCNE2
NCAPG2	CA4	CASS4	RMI2
GINS4	CAB39L	CASZ1	ATAD5
MKI67	CABLES2	CAT	LMNB2
INCENP	CACNA1C	CBFA2T3	TYMS
CHEK1	CACNA2D2	CBX3	AURKB
DSN1	CACNB2	CBX7	KPNA2
NCAPD3	CALCOCO1	CCDC101	TRAIP
CENPU	CANT1	CCDC137	IQGAP3
SPC25	CASC5	CCDC138	KNTC1
SUV39H2	CASKIN2	CCDC167	CDCA2
CENPH	CASP12	CCDC48	WDHD1
SKA1	CAV1	CCDC58	PRC1
EME1	CAV2	CCDC99	CENPW
NUP205	CBFA2T3	CCNA2	FANCG
ATAD2	CBX7	CCNB1	KIFC1
SASS6	CCBE1	CCNB2	UHRF1
BUB1B	CCDC137	CCNE1	

CCNB2	CCDC141	CCNE2
CDC25C	CCDC150	CCNF
MIS18A	CCDC167	CCT2
DONSON	CCDC28B	CCT3
CHAF1B	CCDC34	CCT4
RECQL4	CCDC50	CCT6A
LSM12	CCDC68	CCT7
DBF4B	CCDC69	CCZ1
RACGAP1	CCDC85A	CD52
SPC24	CCHCR1	CD83
C16orf59	CCL23	CDC20
CCNF	CCM2L	CDC25A
KIAA1524	CCNA2	CDC25C
SGOL2	CCNB1	CDC45
GMPS	CCNB2	CDC6
TOPBP1	CCNE1	CDC7
KIF15	CCNE2	CDCA2
RFC4	CCNF	CDCA3
H2AFZ	CCT6A	CDCA4
CDC25A	CD36	CDCA5
POC1A	CD93	CDCA7
HMGB2	CDC20	CDCA8
MAD2L1	CDC25A	CDH5
PTTG1	CDC25C	CDK1
ZNF367	CDC45	CDK4
MELK	CDC6	CDK5RAP1
SKA3	CDCA2	CDKN3
DDIAS	CDCA3	CDT1
LRR1	CDCA4	CEACAM20
CENPN	CDCA5	CEBPZ
MCM7	CDCA7	CENPA
KIAA0101	CDCA8	CENPE
PLK1	CDH19	CENPF
TMEM194A	CDH3	CENPH
RRM1	CDH5	CENPI
CDT1	CDK1	CENPK
CHAF1A	CDK16	CENPL
TK1	CDK4	CENPM
PBK	CDK5	CENPN
FEN1	CDKN1C	CENPO
CKAP2L	CDKN3	CENPW
BUB1	CDO1	CEP152
CDK1	CDT1	CEP55
CDCA4	CEBPD	CEP72

SHCBP1	CELF2	CFP
RRM2	CELSR3	CHAC2
C5orf34	CENPA	CHAF1A
CKS1B	CENPE	CHAF1B
FBXO45	CENPF	CHCHD2
EXO1	CENPH	CHCHD8
ZWILCH	CENPI	CHD9
UBE2C	CENPK	CHEK1
CCNE2	CENPL	CHEK2
RMI2	CENPM	CHRDL1
ATAD5	CENPN	CHRM1
LMNB2	CENPO	CHRNA5
TYMS	CENPU	CHTF18
SUZ12	CENPW	CIRH1A
AURKB	CEP55	CKAP2
CCDC43	CEP85	CKAP2L
GINS3	CERS6	CKAP5
KPNA2	CFD	CKM
SKA2	CFLAR	CKS1B
TRAIP	CHAF1A	CKS2
IQGAP3	CHAF1B	CLCN2
KNTC1	CHD1L	CLDN12
CDCA2	CHEK1	CLDN18
PARPBP	CHEK2	CLEC3B
KIF18B	CHKB-AS1	CLSPN
SAPCD2	CHRDL1	CNO
FANCA	CKAP2	CNPY2
FAM111B	CKAP2L	COASY
TUBB	CKS1B	COL4A3BP
XRCC2	CLDN18	COPS3
BLM	CLDN5	COPS8
PRIM1	CLEC14A	COQ3
WDHD1	CLEC1A	CORO2B
ARHGAP11A	CLIC3	COX15
PRC1	CLIC5	COX4I2
CENPW	CLSPN	COX7A1
C4orf46	CLU	CPAMD8
DNAJC9	CNKSR2	CPSF3
FANCG	CNN1	CPSF6
KIFC1	CNOT11	CRBN
CTD-2510F5.4	CNPY2	CRY2
UHRF1	CNRIP1	CSE1L
	CNTN4	CSH1

CNTN6	CSNK1D
COA4	CSRP1
COA6	CST3
COA7	CST5
COL10A1	CSTF2
COL11A1	CSTF3
COL1A1	CTHRC1
COL3A1	CTIF
COL6A6	CTPS
CORO2B	CTSL2
COX7A1	CYBRD1
CPB2	CYC1
CPED1	CYP27B1
CPSF3	CYP4B1
CRABP2	CYP4Z1
CRBN	CYS1
CREB3L4	DACT3
CRIM1	DAP3
CRTAC1	DARS2
CRYAB	DAZAP1
CSRNP1	DBF4
CSRP1	DCAF13
CST1	DDX11
CTA-445C9.15	DDX27
CTHRC1	DDX39A
CTIF	DDX55
CYBRD1	DDX56
CYP4B1	DEPDC1
CYYR1	DEPDC1B
DAPK2	DES
DARS2	DHFR
DCTPP1	DHX33
DDX5	DIAPH3
DENND2A	DISP1
DENND3	DIXDC1
DEPDC1	DKC1
DEPDC1B	DLGAP5
DES	DNA2
DGKE	DNAJB11
DHFR	DNAJC9
DLC1	DNMT1
DLGAP5	DONSON
DNA2	DPEP2
DNAH14	DPH2

DNAJC22	DPM1
DNAJC27	DPY30
DNASE1L3	DSCC1
DNMT1	DSG2
DNMT3B	DSN1
DNPH1	DTL
DOK6	DTYMK
DOLPP1	DUOX1
DPEP2	DUS1L
DPP3	DUS4L
DPP6	DUSP26
DPYSL2	E2F1
DSCC1	E2F2
DTL	E2F3
DTYMK	E2F5
DUOX1	E2F6
DUOXA1	E2F7
E2F1	E2F8
E2F7	EBNA1BP2
E2F8	ECE2
EARS2	ECT2
ECE2	EDA
ECSCR	EDNRB
ECT2	EEF1E1
EDNRB	EEF2K
EFCAB11	EFTUD2
EFCC1	EIF2C2
EFEMP1	EIF2S2
EFNA3	EIF3B
EFNA4	EIF3I
EGLN3	EIF4A3
EIF1	ELN
EIF4EBP1	EME1
EIF6	EMG1
ELMO1	EMP2
EMC3-AS1	ENO1
EMCN	ENOPH1
EML1	ENTPD7
EMP1	ENY2
EMP2	EPN3
ENC1	EPS15
EPAS1	EPT1
EPB41L4A-AS1	ERCC6L

EPHB2	ERO1L
EPN3	ERVMER34-1
EPT1	ESAM
ERCC6L	ESCO2
ERG	ESPL1
ERH	EXO1
ERO1L	EXOSC1
ESAM	EXOSC5
ESCO2	EXTL3
ESPL1	EZH2
ETV4	F11
EXO1	FAM107A
EXOSC4	FAM111B
EZH2	FAM136A
F10	FAM189A2
F8	FAM208B
FABP4	FAM54A
FADD	FAM64A
FAM105A	FAM72A
FAM107A	FAM72D
FAM110D	FAM83D
FAM13C	FAM91A1
FAM150B	FAM98A
FAM162B	FANCA
FAM167A	FANCB
FAM189A2	FANCD2
FAM195A	FANCG
FAM46B	FANCI
FAM64A	FARSB
FAM83A	FBLN5
FANCD2	FBXO22
FANCG	FBXO5
FANCI	FCER1A
FASTKD1	FEN1
FAT3	FGD5
FBLN5	FGF11
FBXL5	FGFR1OP
FCN3	FHL5
FEN1	FIGF
FENDRR	FIGNL1
FERMT2	FKBP4
FEZ1	FLJ32224
FGD5	FMO2
FGF2	FOLR3

FGR	FOXF1
FHL1	FOXK2
FHL5	FOXM1
FIBIN	FOXRED1
FIGNL1	FRMD5
FILIP1	FTSJ2
FKBP3	FXYD1
FKBPL	FXYD6
FLAD1	G2E3
FLT4	GABPB1
FMO2	GAPDH
FOXF1	GAR1
FOXF2	GARS
FOXM1	GART
FOXO1	GATC
FRMD3	GDF10
FRMD4B	GEMIN6
FRMD5	GEN1
FRY	GFM1
FTSJ2	GGA2
FUT2	GGCT
FUT8	GGH
FXYD1	GINS1
FXYD6	GINS2
FYN	GINS4
FZD4	GJC2
GAB2	GKN2
GABARAPL1	GLRX3
GADD45B	GMCL1
GALNT14	GMNN
GALNT18	GMPS
GALNT6	GNL2
GALNT7	GNL3
GAPDH	GOLT1B
GARS	GORASP2
GAS6	GPD1
GATA2	GPIHBP1
GCNT3	GPM6A
GDF10	GPN1
GEMIN6	GPR146
GFOD1	GPR162
GFRA1	GPR172A
GGCT	GPR19
GHR	GSG2

GIMAP1	GSN
GIMAP6	GSS
GIMAP7	GSTCD
GIMAP8	GSTM5
GINS1	GTF2H3
GINS2	GTF2IRD2
GINS3	GTF3C2
GINS4	GTF3C3
GJB2	GTPBP4
GKN2	GTSE1
GMNN	GYPC
GNAQ	GYPE
GNG11	H2AFV
GOLM1	H2AFX
GPC3	H2AFZ
GPD1	HCCS
GPI	HEATR1
GPIHBP1	HEATR2
GPM6A	HELB
GPM6B	HELLS
GPR133	HIGD1B
GPR146	HIST1H1B
GPRASP1	HIST1H2AB
GPRC5A	HIST1H2AG
GPT2	HIST1H2AI
GPX3	HIST1H2AL
GREM1	HIST1H2AM
GRIA1	HIST1H2BB
GRK5	HIST1H2BC
GSS	HIST1H2BD
GSTM5	HIST1H2BG
GTF3C6	HIST1H2BI
GTSE1	HIST1H2BJ
GUCY1A2	HIST1H2BM
GYLTL1B	HIST1H3B
GYPC	HIST1H3G
HBB	HIST1H3H
HECW2	HIST1H3I
HEG1	HIST1H4F
HELLS	HIST2H3A
HES6	HIST2H3D
HHIP	HJURP
HIGD1B	HLA-DMA
HIST1H2BD	HLF

HIST1H2BH	HLTF
HIST1H4J	HLX
HJURP	HM13
HLA-E	HMBS
HMBOX1	HMGA1
HMBS	HMGB2
HMGB3	HMGXB4
HMGB3P1	HMMR
HMGCLL1	HN1
HMMR	HOXA5
HN1	HPDL
HN1L	HPGDS
HNRNPA3	HPRT1
HOXA4	HPS3
HOXB7	HSF2BP
HS6ST2	HSPA12B
HSD17B10	HSPB2
HSD3B7	HSPD1
HSF2BP	HSPE1
HSPA12B	HYLS1
HSPB2	IARS
HSPB8	ICT1
HUS1	IGF2BP3
HYAL1	IHH
HYAL2	IL33
ICAM2	ILF2
ICMT	INCENP
IDH2	INMT
IFT88	INPP5K
IGF2BP3	INTS2
IGFL2	INTS7
IGSF10	INTS8
IGSF9	IPO4
IKBKE	IQGAP3
IL33	ITGA8
ILF2	ITIH3
INMT	JPH4
INPP5K	KANK2
INTS8	KANK3
IPO4	KDELR2
IQGAP3	KHDC1
ISCA2	KIAA0090
ITGA11	KIAA0101
ITGA8	KIAA1462

ITIH5	KIF11
ITM2A	KIF14
ITPR1	KIF15
JADE1	KIF17
JAM2	KIF18A
JDP2	KIF1C
JTB	KIF20A
KAL1	KIF20B
KANK2	KIF22
KANK3	KIF23
KAT2B	KIF24
KAT6A	KIF2A
KCNK3	KIF2C
KCNN4	KIF4A
KCNT2	KIFC1
KCTD10	KLF2
KDELR2	KLHL7
KDM1A	KNTC1
KIAA0101	KPNA2
KIAA1377	KPNB1
KIAA1462	KRT80
KIF11	LBH
KIF14	LCTL
KIF15	LDB2
KIF17	LDHA
KIF18B	LEPREL4
KIF1C	LHFP
KIF20A	LIG1
KIF23	LIMS2
KIF26B	LINC00312
KIF2C	LINC00341
KIF4A	LINC00472
KIFC1	LLPH
KISS1R	LMBR1
KL	LMBRD1
KLF2	LMNB1
KLF4	LMNB2
KLF6	LMO2
KLF9	LMOD1
KNOP1	LOC100128881
KNTC1	LOC100131347
KPNA2	LOC100132724
KRT10	LOC100188947
L2HGDH	LOC100286979

LAD1	LOC100287063
LAGE3	LOC100287852
LAMP3	LOC100288637
LATS2	LOC100289092
LDB2	LOC100294338
LDHA	LOC100505692
LEPRE1	LOC100505938
LEPREL4	LOC100505976
LEPROT	LOC100506303
LHFP	LOC100506542
LIFR	LOC100507212
LIG1	LOC100507312
LIMCH1	LOC100507632
LIMK1	LOC100652786
LIMS2	LOC286437
LIN7A	LOC339524
LINC00312	LOC389033
LINC00467	LOC399815
LINC00472	LOC400550
LINC00702	LOC400568
LINC00968	LOC400950
LINC01140	LOC645722
LMNB1	LOC723809
LMNB2	LOC727803
LMO2	LOC728392
LMO7	LOC729313
LMOD1	LOC81691
LNP1	LRIF1
LOC100506725	LRPPRC
LOC100506990	LRR1
LOC100996760	LRRC18
LOC101927929	LRRC36
LOC101928370	LRRC42
LOC153577	LRRC59
LOC285043	LRRN3
LOC285812	LSM12
LONRF1	LSM5
LPL	LST1
LRR1	LTBP4
LRRC15	LTC4S
LRRC32	LY86
LRRC8D	LYPLA1
LRRK2	MACF1
LRRN3	MACROD2

LSAMP	MAD2L1
LSR	MAGOHB
LTBP4	MAMDC2
LYPD1	MAOB
LYVE1	MAP3K3
MAD2L1	MAP6
MAFG-AS1	MAP6D1
MAGED1	MAPKAPK5
MAL	MARS2
MAMDC2	MAST4
MANEAL	MASTL
MANF	MCFD2
MAOB	MCM10
MAP3K3	MCM2
MAP3K8	MCM3
MAPK13	MCM4
MARCKSL1	MCM5
MARCO	MCM6
MARVELD3	MCM7
MASP1	MCM8
MBNL1-AS1	MDH2
MCEMP1	MED10
MCM10	MEF2A
MCM2	MELK
MCM3	MEMO1
MCM4	MEST
MCM5	METTL1
MCM6	METTL23
MCM8	METTL5
MDK	METTL7A
MEA1	MFAP4
MECP2	MFNG
MEIS1	MGAT3
MELK	MGP
MEX3A	MIF
MFAP4	MINA
MFI2	MINPP1
MIF	MIRLET7BHG
MIS18A	MIS18A
MKI67	MKI67
MLEC	MKI67IP
MME	MLF1IP
MMP1	MMP12
MMP11	MMP28

MMP12	MMS22L
MMP13	MND1
MMP14	MOGAT1
MMRN1	MPHOSPH9
MMRN2	MPP6
MND1	MRPL1
MOCS1	MRPL12
MPDZ	MRPL13
MRPL12	MRPL15
MRPL17	MRPL17
MRPL24	MRPL19
MRPL27	MRPL3
MRPL9	MRPL36
MRPS16	MRPL38
MRPS17	MRPL47
MRPS34	MRPL9
MS4A15	MRPS12
MSI2	MRPS17
MSN	MRPS23
MSTO1	MRPS24
MT1M	MRPS30
MTFP1	MRPS33
MTFR2	MRPS7
MTHFD1	MRTO4
MTIF2	MSH2
MTURN	MSH6
MTX1	MSI2
MYADM	MSTO1
MYBL2	MTA3
MYCT1	MTBP
MYH10	MTFP1
MYH11	MTFR1
MYLK	MTHFD1
MYO19	MTHFD1L
MYOCD	MTHFD2
MYZAP	MTMR10
NCAPD2	MTX2
NCAPD3	MUSTN1
NCAPG	MYBL2
NCAPG2	MYH10
NCAPH	MYH11
NCKAP5	MYL9
NCOA1	MYO19
NDC1	MYO9A

NDC80	MYOC
NEBL	MYOCD
NEDD4L	MYOZ3
NEDD9	MYZAP
NEIL3	N4BP1
NEK2	N4BP2L2
NFASC	NAA15
NIPSNAP1	NAA25
NKIRAS2	NAP1L4
NLN	NAT9
NME1	NCAPD2
NMRAL1	NCAPD3
NMU	NCAPG
NOSTRIN	NCAPG2
NOTCH4	NCKAP5
NPM3	NCL
NPNT	NDC80
NPR1	NDRG2
NR2F1	NDST1
NRG3	NDUFB9
NTN4	NDUFS6
NTNG1	NEK2
NTRK3	NEXN
NUDT1	NFIX
NUF2	NFS1
NUP210	NIF3L1
NUP85	NLE1
NUPL2	NLN
NUSAP1	NME1
OCIAD2	NME2
OGN	NMU
OIP5	NOC3L
OLFML1	NOL10
OR7E47P	NOL11
ORC1	NOLC1
ORC6	NOP14
OTUB2	NOP16
OTUD1	NOP2
P2RY14	NOTCH4
P4HA3	NPR1
PAFAH1B3	NR2F1
PAICS	NSUN2
PALMD	NT5C3L
PAPSS2	NTRK3

PAQR4	NUDT1
PARP1	NUDT5
PARP2	NUF2
PARPBP	NUP107
PARVA	NUP155
PBK	NUP205
PCAT19	NUP210
PCAT6	NUP37
PCNA	NUP85
PCOLCE2	NUPR1
PCP4	NUSAP1
PDCD2L	NXF3
PDE2A	NXPH3
PDE8B	OBFC2B
PDIA4	OGN
PDK1	OIP5
PDK4	ORC1
PDLIM2	ORC5
PDZD11	ORC6
PDZD2	OSTC
PEAK1	OSTCP1
PEAR1	OSTCP2
PEBP4	P4HB
PECAM1	PA2G4
PGM2L1	PAICS
PGM5	PALMD
PGM5-AS1	PAQR4
PGP	PARG
PHACTR1	PARP1
PHF19	PAXIP1
PHF6	PBK
PIAS1	PCBD2
PID1	PCDP1
PIGU	PCF11
PIK3C3	PCGF6
PIK3R2	PCM1
PIP5K1B	PCNA
PKIG	PDCD11
PKNOX2	PDCD2L
PLAC8	PDCD5
PLAC9	PDE5A
PLCL1	PDE8B
PLEK2	PDIA4
PLEKHA8	PDIA6

PLEKHH2	PDRG1
PLK1	PDSS1
PLK4	PDZD11
PLLP	PDZD2
PLOD1	PEAK1
PLOD2	PEBP4
PLSCR4	PECAM1
POC1A	PFDN2
PODXL	PFDN4
POLD2	PFKP
POLDIP2	PFN2
POLE2	PGAM1
POLQ	PGM3
POLR2H	PHACTR1
PPAP2C	PHB
PPAT	PHF19
PPIL1	PHF2
PPM1G	PI16
PPME1	PIF1
PPP1R14A	PIGW
PPP1R14B	PKMYT1
PPP1R15A	PLA2G1B
PRC1	PLAC1
PRDM11	PLAC8
PRDX4	PLAC9
PRELP	PLK4
PRICKLE2	PNO1
PRKCH	PNPT1
PRMT5	POC1A
PROM2	PODXL2
PRX	POLA2
PSAT1	POLD1
PSMB3	POLD2
PSMB4	POLDIP2
PSMB5	POLE2
PSMC1	POLE3
PSMD3	POLG2
PSMD4	POLQ
PSMG3	POLR1B
PTGDS	POLR2D
PTGFRN	POP1
PTH1R	POP7
PTK7	PPAT
PTPN21	PPIA

PTPRB	PPIF
PTPRM	PPIL1
PTRF	PPM1G
PTTG1	PPP1R35
PTTG3P	PRAM1
PYCR1	PRC1
PZP	PRDM6
QKI	PRICKLE1
R3HDM1	PRIM1
RAB11FIP1	PRKDC
RAB37	PRMT3
RABIF	PRPF4
RAC3	PRPS2
RACGAP1	PRR11
RAD51	PRR19
RAD51AP1	PRR3
RAD54L	PRTFDC1
RAI2	PSAT1
RAMP2	PSMA2
RAMP3	PSMB2
RAP1A	PSMB3
RAPGEF4	PSMB4
RASIP1	PSMC3IP
RASL12	PSMC4
RASSF2	PSMD1
RBMS2	PSMD11
RBMS3	PSMD12
RBP4	PSMD14
RCC1	PSMD2
RCC2	PSMG3
RCCD1	PSRC1
RDM1	PTCRA
RECQL4	PTGDS
RFC2	PTGES2
RFC4	PTPN21
RGCC	PTRH2
RHNO1	PTS
RHOJ	PTTG1
RILPL2	PTTG2
RMI2	PTTG3P
RNASEH2A	PUF60
RNF144B	PURA
RNF182	PUS1
RNFT2	PUS7

ROBO2	PYCR1
ROBO4	PYCRL
RP1-78O14.1	Q9B8G3
RP11-1024P17.1	QSOX2
RP11-127B20.2	R3HDM1
RP11-295M18.6	RAB44
RP11-389C8.2	RACGAP1
RP11-401P9.4	RACGAP1P
RPA3	RAD1
RPL39L	RAD18
RPN2	RAD51
RPUSD1	RAD51AP1
RRAS	RAD51C
RRM1	RAD54B
RRM2	RAD54L
RSPO1	RAI2
RTKN2	RAMP2
RUNX2	RAN
RUSC1	RANBP1
RXFP1	RBL1
S1PR1	RCC1
SAC3D1	RCCD1
SACM1L	RECQL4
SAP18	RECQL5
SAPCD2	REEP5
SASH1	RETN
SBF2-AS1	RFC2
SBK1	RFC3
SCAI	RFC4
SCARA5	RFC5
SCG5	RFWD3
SCGB1A1	RFX2
SCGB3A2	RHOJ
SCN2B	RILPL2
SCN4B	RMI2
SCN7A	RNASEH1
SCNN1G	RNASEH2A
SDF2L1	RNF180
SDPR	RNFT2
SEC61G	ROBO2
SELP	RPA3
SEMA3G	RPAP3

SEMA5A	RPE
SEMA6A	RPL13AP17
SEMA6D	RPL39L
SEPP1	RPLP0
SERINC2	RPP40
SERTM1	RPS21
SESN1	RPS7
SETBP1	RRM1
SFTA1P	RRM2
SFTPC	RRP1B
SFTPD	RRP36
SFXN1	RRS1
SFXN4	RSRC1
SGCA	RTKN2
SGCG	RUNX1T1
SGOL1	RUVBL1
SGOL2	RXRA
SGPL1	S1PR4
SH2D3C	SAAL1
SH3BP5	SASS6
SH3GL2	SCARA5
SH3GL3	SCN2B
SHANK3	SCN4B
SHCBP1	SCN7A
SHFM1	SCRIB
SHMT2	SDCCAG3
SHROOM4	SDPR
SIK2	SEC14L3
SKA1	SEC61G
SKA3	SEMA3G
SLC19A3	SEMA5A
SLC1A4	4-Sep
SLC25A10	SF3B14
SLC25A13	SFTA1P
SLC25A39	SFXN4
SLC2A1	SGCA
SLC39A7	SGCG
SLC46A2	SGOL1
SLC50A1	SGOL2
SLC6A4	SH2D3C
SLC6A8	SHC3
SLCO5A1	SHCBP1
SLFN13	SIGLECP3
SLIT2	SKA1

SLIT3	SKA2
SLK	SKA3
SMAD9	SLBP
SMCO3	SLC16A3
SMYD3	SLC19A3
SNCA	SLC25A10
SNRK	SLC25A13
SNRNP25	SLC25A15
SNRPC	SLC25A39
SNX1	SLC27A1
SNX6	SLC2A1
SOCS2	SLC30A6
SORBS1	SLC35B1
SOSTDC1	SLC35B2
SOX17	SLC50A1
SOX4	SLC6A16
SOX7	SLC6A4
SPAG4	SLC7A1
SPAG5	SLC7A5
SPARCL1	SLFNL1
SPATS2	SLIT2
SPC24	SMAD6
SPC25	SMARCA2
SPDL1	SMC2
SPG20	SMC4
SPNS2	SMUG1
SPOCK2	SNF8
SPP1	SNHG1
SPTBN1	SNORA56
SPX	SNORD18C
SRD5A1	SNORD4B
SRM	SNORD70
SRPK1	SNORD71
SRPRB	SNORD72
SRPX	SNORD86
SSR4	SNORD91A
ST14	SNRPA1
ST6GALNAC6	SNRPD1
STAP2	SNRPD2
STARD13	SNRPD2P2
STARD8	SNRPD3
STARD9	SNRPF
STIL	SNRPG
STK39	SNX22

STMN1	SNX30
STOM	SPAG4
STRA13	SPAG5
STX11	SPARCL1
STX12	SPATA18
STXBP6	SPC24
STYXL1	SPC25
SULF1	SPOCK2
SUV39H2	SPTAN1
SVEP1	SRD5A1
SYNE1	SRP68
SYNM	SRPRB
SYNPO2	SRSF9
TACC1	SSRP1
TACC3	ST6GALNAC6
TACO1	STARD13
TAL1	STEAP1
TBX2	STEAP1B
TBX3	STIL
TBX5	STIP1
TBX5-AS1	STRA13
TCF19	STX12
TCF21	SULT1A2
TCF3	SUV39H1
TDP1	SUV39H2
TEK	SUZ12
TENC1	SYAP1
TENM4	SYNE1
TFAP2A	SYNPO2
TGFBR3	SYT15
THBD	TACC1
THBS2	TACC3
TICRR	TACO1
TIE1	TADA2A
TIMELESS	TAF2
TIMM50	TARS
TIMM9	TATDN1
TIMP3	TBRG4
TK1	TBX2
TLCD1	TBX3
TMED3	TBX4
TMED9	TCEB1
TMEM100	TCF19
TMEM177	TCF21

TMEM178A	TDG
TMEM184A	TDRKH
TMEM204	TEK
TMEM47	TENC1
TMEM63C	TFCP2
TMEM74B	TGFB1I1
TMEM88	THOC4
TMOD1	THOP1
TMPO-AS1	THSD1
TMTC1	TIGD5
TNFRSF18	TIMELESS
TNNC1	TIMM23
TNPO1	TIMM50
TNS1	TIMM8A
TOX2	TIPIN
TPBG	TK1
TPI1	TLR5
TPX2	TMEM100
TRAF4	TMEM132A
TRAIP	TMEM167A
TRHDE	TMEM177
TRIM59	TMEM182
TRIP13	TMEM194A
TRMT5	TMEM199
TROAP	TMEM206
TRPC6	TMEM223
TSC22D1	TMEM48
TSPAN18	TMEM69
TSPAN5	TMEM79
TSPAN7	TMPO
TTC28	TNFSF12
TTK	TNNC1
TUBB	TNS1
TUBG1	TNXB
TXNDC17	TOMM40
TYMS	TOMM70A
UBE2C	TONSL
UBE2T	TOPBP1
UBL3	TPI1
UGGT1	TPI1P2
UHRF1	TPPP3
UPK3B	TPRKB
UQCRQ	TPX2
UROS	TRAIP

USP53	TRIM37
VAPA	TRIM59
VARS	TRIP13
VDR	TRMT5
VGLL3	TROAP
VIPR1	TSFM
VPS72	TSN
VWF	TSR1
WASF3	TTC27
WDHD1	TTF2
WFDC1	TTI1
WFS1	TTK
WHSC1	TUBA1C
WIF1	TUBB3
WISP2	TUBG1
WNT2B	TWF1
XDH	TYMS
XPO5	UBA2
XPR1	UBE2C
YDJC	UBE2S
YIPF1	UBE2T
YIPF2	UBE3C
YKT6	UBFD1
YPEL5	UCHL5
ZBTB16	UCK2
ZBTB4	UHRF1
ZFP36	UMPS
ZNF106	UNG
ZNF93	UNK
ZWINT	UPK3B
	URB2
	USHBP1
	USP39
	UTP11L
	UTP18
	UTP6
	VGLL3
	VIPR1
	VRK1
	VSIG2
	WDHD1
	WDR12
	WDR3
	WDR4

WDR46
WDR62
WDR7
WDR75
WDYHV1
WHSC1
WNT3A
WRN
XLOC_001035
XLOC_001043
XLOC_001412
XLOC_002211
XLOC_003406
XLOC_006043
XLOC_006099
XLOC_006994
XLOC_008390
XLOC_008711
XLOC_008730
XLOC_008855
XLOC_009724
XLOC_011535
XLOC_011872
XLOC_013194
XLOC_l2_003944
XLOC_l2_004222
XLOC_l2_005039
XLOC_l2_008275
XLOC_l2_008289
XLOC_l2_009029
XLOC_l2_009623
XLOC_l2_010149
XLOC_l2_010228
XLOC_l2_013460
XLOC_l2_013480
XLOC_l2_014191
XLOC_l2_014504
XLOC_l2_015451
XLOC_l2_015738
XPO1
XPOT
XRCC3
XYLB
YARS

YDJC
YEATS4
YKT6
YPEL5
YWHAZ
ZBTB4
ZBTB47
ZC3HC1
ZFP106
ZHX1-C8ORF76
ZMYND15
ZNF146
ZNF204P
ZNF207
ZNF259
ZNF259P1
ZNF367
ZNF485
ZNF544
ZNF607
ZNF695
ZNHIT3
ZWILCH
ZWINT
ZYG11A
