# Supplementary material for: The role of TOP2A in immunotherapy and vasculogenic mimicry in non-small cell lung cancer and its potential mechanism
Source: Sci Rep. 2023 Jul 5;13:10906. doi: 10.1038/s41598-023-38117-6 (PMC10322841; doi:10.1038/s41598-023-38117-6)
Supplement: Supplementary file 8 — Supplementary Table S3. [file 41598_2023_38117_MOESM8_ESM.pdf]

Table S3: The correlation between VM and clinicopathological characteristics in NSCLC

Variables	VM		P
	Negative	Positive	
Age (years)			0.096
< 60	44	21	
⩾60	41	35	
Gender			0.069
Female	42	19	
Male	43	37	
Smoking			0.453
No	54	39	
Yes	31	17	
Gross Type			0.837
Central	44	28	
Peripheral	41	28	
Histologic Type			0.422
SCC	11	10	
Ade	74	46	
Grade			0.008
Well	22	8	
Moderate	52	29	
Poor	11	19	
LNM			0.002
No	57	23	
Yes	28	33	
TNM stage			0.024
Ⅰ	43	18	
Ⅱ	22	13	
Ⅲ	20	25	
