# Supplementary material for: The role of TOP2A in immunotherapy and vasculogenic mimicry in non-small cell lung cancer and its potential mechanism
Source: Sci Rep. 2023 Jul 5;13:10906. doi: 10.1038/s41598-023-38117-6 (PMC10322841; doi:10.1038/s41598-023-38117-6)
Supplement: Supplementary file 9 — Supplementary Table S4. [file 41598_2023_38117_MOESM9_ESM.pdf]

Table S4: Correlation of TOP2A expression pattern and tumor cytoskeleton and motility.

Data source	Gene Set Name	Size	NES	NOM P-val	FDR q-val
TCGA-LUAD	GOBP_CYTOSKELETON_DEPENDENT_CYTOKINESIS	98	2.42	0.00	0.00
	GOBP_MICROTUBULE_CYTOSKELETON_ORGANIZATION_INVOLVED_IN_MITOSIS	157	2.42	0.00	0.00
	GOBP_PROTEIN_LOCALIZATION_TO_CYTOSKELETON	58	2.28	0.00	0.00
	GOBP_REGULATION_OF_MICROTUBULE_CYTOSKELETON_ORGANIZATION	151	2.14	0.00	0.00
	GOBP_CORTICAL_ACTIN_CYTOSKELETON_ORGANIZATION	39	1.74	0.03	0.04
GSE116959	GOBP_MICROTUBULE_CYTOSKELETON_ORGANIZATION_INVOLVED_IN_MITOSIS	149	1.89	0.00	0.01
	GOBP_CYTOSKELETON_DEPENDENT_CYTOKINESIS	95	1.75	0.00	0.02
	GOBP_PROTEIN_LOCALIZATION_TO_CYTOSKELETON	53	1.72	0.01	0.02
	GOBP_REGULATION_OF_MICROTUBULE_CYTOSKELETON_ORGANIZATION	148	1.43	0.04	0.15
GSE11969	GOBP_CYTOSKELETON_DEPENDENT_CYTOKINESIS	92	1.95	0.00	0.00
	GOBP_PROTEIN_LOCALIZATION_TO_CYTOSKELETON	58	1.86	0.00	0.01
	GOBP_MICROTUBULE_CYTOSKELETON_ORGANIZATION_INVOLVED_IN_MITOSIS	149	1.85	0.00	0.01
	GOBP_REGULATION_OF_MICROTUBULE_CYTOSKELETON_ORGANIZATION	143	1.72	0.00	0.02
